# Ligand-mediated and tertiary interactions cooperatively stabilize the P1 region in the guanine-sensing riboswitch

**DOI:** 10.1371/journal.pone.0179271

**Published:** 2017-06-22

**Authors:** Christian A. Hanke, Holger Gohlke

**Affiliations:** Mathematisch-Naturwissenschaftliche Fakultät, Institut für Pharmazeutische und Medizinische Chemie, Heinrich-Heine-Universität Düsseldorf, Düsseldorf, Germany; Computational Biophysics, GERMANY

## Abstract

Riboswitches are genetic regulatory elements that control gene expression depending on ligand binding. The guanine-sensing riboswitch (Gsw) binds ligands at a three-way junction formed by paired regions P1, P2, and P3. Loops L2 and L3 cap the P2 and P3 helices and form tertiary interactions. Part of P1 belongs to the switching sequence dictating the fate of the mRNA. Previous studies revealed an intricate relationship between ligand binding and presence of the tertiary interactions, and between ligand binding and influence on the P1 region. However, no information is available on the interplay *among* these three main regions in Gsw. Here we show that stabilization of the L2-L3 region by tertiary interactions, and the ligand binding site by ligand binding, cooperatively influences the structural stability of terminal base pairs in the P1 region in the presence of Mg^2+^ ions. The results are based on molecular dynamics simulations with an aggregate simulation time of ~10 μs across multiple systems of the unbound state of the Gsw aptamer and a G37A/C61U mutant, and rigidity analyses. The results could explain why the three-way junction is a central structural element also in other riboswitches and how the cooperative effect could become contextual with respect to intracellular Mg^2+^ concentration. The results suggest that the transmission of allosteric information to P1 can be entropy-dominated.

## Introduction

Riboswitches are genetic regulatory elements that regulate gene expression on the RNA level. Most riboswitches are found in the 5'-untranslated region of bacterial mRNA [1,2]. Riboswitches typically consist of an aptamer domain and an expression platform. The former binds ligands with high specificity [3]; the latter undergoes a conformational change upon binding of a ligand in the aptamer domain. Two prevalent modes of gene regulation by riboswitches were found in bacteria: translational and transcriptional gene regulation [4]. In transcriptionally acting riboswitches, the expression platform is involved in the formation of an intrinsic transcription terminator or antiterminator [5]. For this type of gene regulation to be effective, a decision at a branch point during transcription has to be made in favor of one of these two folding pathways [6,7]. Therefore, the unbound state has to maintain ligand-binding competence, but at the same time has to be able to follow the alternative pathway in the absence of the ligand [6].

**Fig 1:**
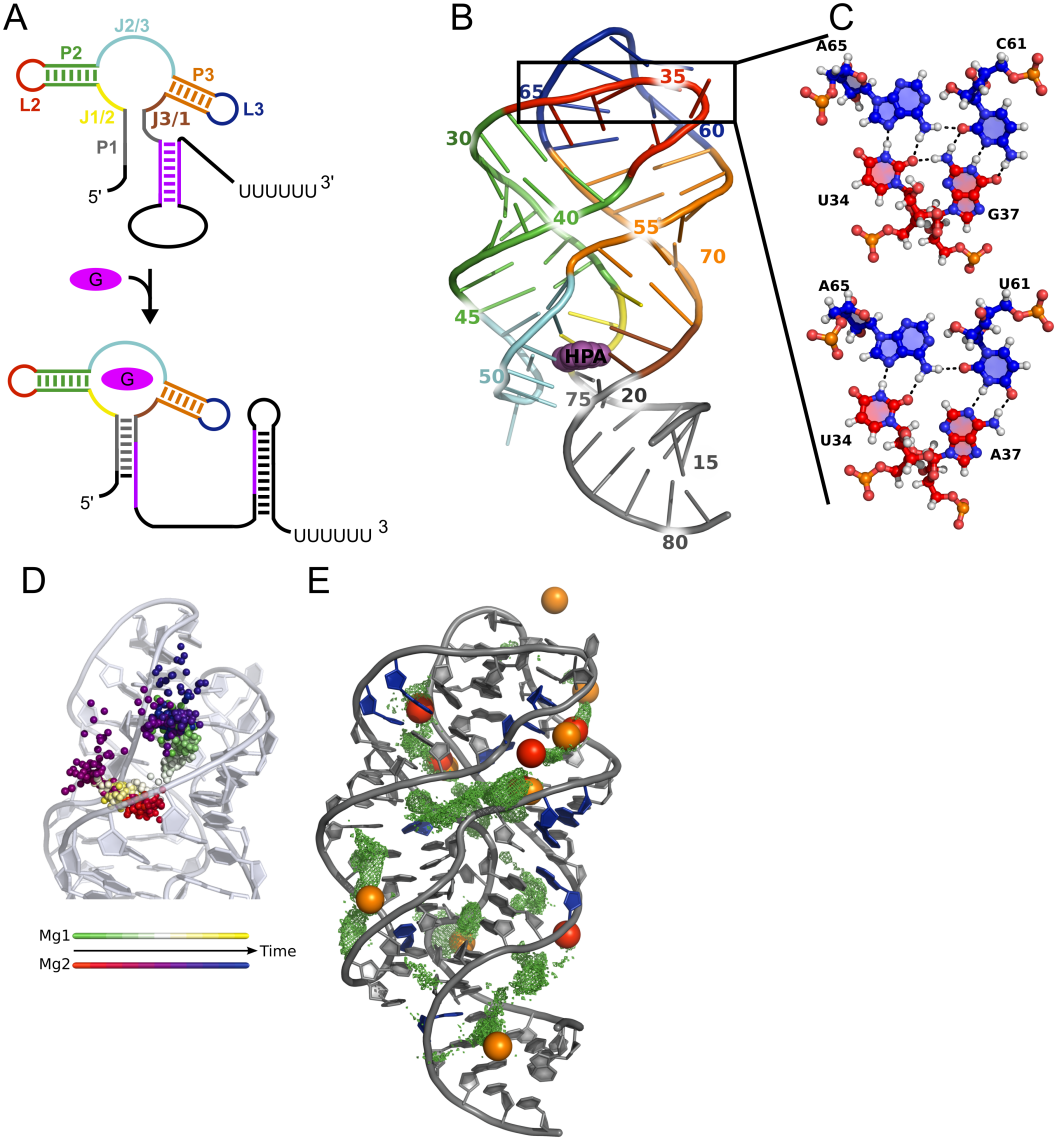
Structural features of the guanine-sensing riboswitch aptamer domain and behavior of Mg^2+^ ions. A: Schematic view of transcriptional regulation by the guanine-sensing riboswitch. In the unbound state (top), the switching sequence (purple) is involved in the formation of the anti-terminator. In the guanine (purple oval) bound state (bottom), the P1 region is stabilized, and part of the switching sequence is involved in the formation of the transcription terminator loop. Regions are assigned according to ref. [8]; grey: P1, green: P2, orange: P3, red: L2, blue: L3, yellow: J1/2, cyan: J2/3, brown: J3/1. B: Tertiary structure of the guanine-sensing riboswitch bound to hypoxanthine (HPA in purple) (PDB ID 4FE5) colored according to secondary structure elements as in panel A; the box marks the mutation site. C: Difference between Gsw^apt^ (top) and Gsw^loop^ (bottom) in the loop region. The G37A/C61U mutation results in a loss of two hydrogen bonds. Bases are colored as in panels A and B according to which loop they belong to. D: Exchange of two Mg^2+^ ions over a time of 8 ns. The positions of the two Mg^2+^ ions are shown as spheres and colored according to time with two different color scales. E: Comparison of preferred sites of occupancy of Mg^2+^ ions during 550 ns of MD simulations (green) to experimentally determined ion binding sites (red/orange: binding sites of [Co(NH_3_)_6_]^3+^ ions in X-ray structures with PDB ID 4FE5 [17]/3RKF [18]) and nucleotides showing chemical shift changes upon magnesium titration in NMR experiments (blue) [18].

The guanine-sensing riboswitch (Gsw) from the *xpt-pbuX* operon of *Bacillus subtilis* investigated here belongs to the class of purine binding riboswitches, binds guanine and hypoxanthine and acts transcriptionally [8,9]. The aptamer domain of this riboswitch consists of three paired regions P1, P2, and P3, two loops L2 and L3 capping the P2 and P3 helices, respectively, and three joining regions J1/2, J2/3 and J3/1 connecting the paired regions [8] (Fig 1A). Part of the P1 region belongs to the switching sequence, which is involved in the formation of the alternative structural element as a consequence of ligand binding [10]. Tertiary interactions between bases from L2 and L3 involve two base quadruples, forming a hydrogen bond network and keeping the P2 and P3 helices in close contact (Fig 1B and 1C). This arrangement of P2 and P3 together with P1 forms a three-way junction around J1/2, J2/3, and J3/1, which is a recurrent structural element in several RNA structures, including other riboswitches [11–13]. Ligands bind to Gsw’s aptamer domain via hydrogen bond interactions with the joining regions J1/2, J2/3 and J3/1. C74 from the J3/1 region is responsible for ligand selectivity of this riboswitch [9]. The ligand is deeply buried in the binding site, which indicates that local flexibility is required for the ligand to bind [8]. J2/3, due to its comparatively high flexibility in the unbound state, is assumed to allow access of the ligand to the binding site [9,14,15]. In order to influence the gene regulation, the information about ligand binding has to be transmitted to the switching sequence in the P1 region. This communication may occur by base stacking interactions of the ligand with nucleotides of the P1 region [8] or by the stabilization of base triples between nucleotides of the J2/3 and P1 regions [16].

The tertiary L2-L3 interactions were found to play an important supporting role in facilitating ligand binding [16], especially of lower-affinity ligands such as hypoxanthine [8,19]. The replacement of the loops by stable UUCG tetraloops [8] resulted in a mutant that did not bind hypoxanthine. Although mutations outside the base quadruples were found to not significantly affect ligand binding [20], mutations in the base quadruples are less tolerated [20]. A G37A mutation resulted in an aptamer which did not bind hypoxanthine but was still able to bind guanine [16]. The additional mutation of C61U yielded a mutant G37A/C61U in which two of the seven hydrogen bonds in the respective wildtype base quadruple are missing. This double mutant showed a Mg^2+^ dependence of the ability to bind hypoxanthine [15]. It only regains the ligand binding ability in the presence of Mg^2+^ ions at a [Mg^2+^]:[RNA] ratio > 18:1 [15,18]. This makes the double mutant an interesting test system where the stability of the tertiary interactions, and its influence on ligand binding, can be fine-tuned via the Mg^2+^ concentration. In the opposite direction, time-resolved NMR investigations using a photo-caged ligand showed that ligand binding stabilizes the L2-L3 interactions [14]. Furthermore, nonequilibrium MD simulations of temperature-induced energy flow in the Gsw reported a communication between the nucleotides at the ligand binding site and the distant L2-L3 region [21]. Additionally, umbrella sampling simulations using the distance between the loops as a reaction coordinate were applied to investigate the influence of the ligand on the formation of the loop-loop interactions in an adenine-sensing riboswitch [22,23]. Similarly, ligand-induced stabilization of the P1 region was investigated using steered molecular dynamics simulations, where the strands of the P1 region were separated by force [24]. An NMR study on an adenine-sensing riboswitch observed a coupling of the stability of the P1 region and the tertiary interactions with the ligand binding ability [25]. However, this effect was suggested to be smaller for the more stable guanine-sensing riboswitch aptamer [25].

Detailed structural information of the ligand-bound state of riboswitch aptamer domains is provided by crystal structures, including those of Gsw [8,26], and other experimental and theoretical investigations (reviewed in refs. [10,11,27,28]). In contrast, the unbound state is by far less well characterized, especially at the atomistic level. Although crystal structures of unbound riboswitch aptamer domains other than from Gsw have been resolved [29], they only provide static information for what is considered a highly dynamic state [6,10,30]. In the case of Gsw, NMR investigations, in-line probing, and SHAPE experiments showed that the global fold of the unbound state of the aptamer domain is likely very similar to the bound state and is usually described as largely preorganized but locally disordered, with the P1, P2, and P3 regions preformed [31,32]; NMR studies revealed that this also holds even in the absence of Mg^2+^ ions [31]. In contrast, the ligand binding core, formed by J1/2, J2/3, and J3/1, is generally assumed to be disorganized [9,16,31,32]. The tertiary L2-L3 interactions are typically described as preformed in the unbound state even in the absence of Mg^2+^ ions [11,18,31]. However, the existence of a population of structures lacking the tertiary interactions is discussed [19,32]. A FRET study proposed that in the absence of Mg^2+^ and ligand the aptamer domain contains a highly dynamic P2 helix that moves with respect to the P3 helix, thereby allowing to form only transient L2-L3 interactions [19]. In the presence of Mg^2+^, however, the studies agree that Gsw’s L2-L3 interactions are stably formed [10]. For the G37A/C61U mutant, the loop-loop interactions were found to be formed only at a [Mg^2+^]:[RNA] ratio > 18:1 [15,18].

From the above, an intricate picture emerges on the relationship between ligand binding to the aptamer domain of Gsw and presence of tertiary L2-L3 interactions, as well as between ligand binding and influence on the P1 region. However, to the best of our knowledge, no atom-level information is available on the interplay *among* these three main regions in Gsw, the L2-L3 region, the ligand binding core, and the P1 region.

Here, we investigate the interplay among these three regions by molecular dynamics (MD) simulations with an aggregate simulation time of ~10 μs of two variants of Gsw’s aptamer domain: the wild type (Gsw^apt^) and the G37A/C61U mutant (Gsw^loop^). Based on a previous study by us [33], we pay particular attention to the force field influence and the influence of modeling Mg^2+^ on the structural dynamics of these RNAs. Finally, we perform rigidity analyses on the MD ensembles, which reveals that L2-L3 and ligand-mediated interactions cooperatively stabilize the terminal nucleotides in the P1 region, even in the absence of conformational changes in the binding site. We then discuss how our functional insights may provide an explanation as to why the three-way junction is a central structural element also in riboswitches, how the cooperative stabilization could become contextual with respect to the intracellular Mg^2+^ concentration, and what significance our findings have for the full aptamer’s switching function.

## Results

For unbound Gsw^apt^ and Gsw^loop^ with 0, 12, and 20 Mg^2+^ ions in the simulation box, three independent MD simulations of 550 ns length each were performed. Unless otherwise noted, results of these 18 MD simulations are reported below.

### Mg^2+^ ions show high initial mobility and occupy binding sites in good agreement with experimentally determined ones

Mg^2+^ ions have a strong influence on structure and stability of RNA [34,35]. However, modeling these ions in MD simulations is considered difficult [36] because Mg^2+^ ions show a slow exchange kinetics of first shell ligands [37] and display very slow diffusion rates [38,39]. Hence, given the length of our MD simulations of 550 ns, it was important to carefully place the Mg^2+^ ions in the simulation box to prevent poor sampling. To account for this, we placed the Mg^2+^ ions as hexahydrated complexes at electrostatically favorable positions along the RNA. For details about this placement procedure and its advantages over placing non-hydrated Mg^2+^, see ref. [33].

As a result, more than half of the Mg^2+^ ions showed a pronounced mobility during the first ~100 ns of the MD simulations (see Figure 2 in ref. [33]), and several ions remained mobile even after 500 ns. Additionally, we observed that several of the ions explore a large region around the aptamer in the MD simulations (shown exemplarily for three ions in S1 Fig). Furthermore, we occasionally observed that (temporarily) immobile hexahydrated Mg^2+^ ions swapped their positions during the MD simulations (Fig 1D). For other immobile Mg^2+^ ions, we observed that the ions lost water molecules from their first hydration shell and started to interact with the RNA (S2 Fig), despite the slow exchange kinetics. These observations are consistent with the known bias towards inner-shell binding of Mg^2+^ ions in RNA simulations using a non-polarizable force field [36,40,41]. S2 Fig also reveals that for both Gsw^apt^ and Gsw^loop^ with 12 Mg^2+^ and Gsw^apt^ with 20 Mg^2+^ the decrease in the number of hexahydrated Mg^2+^ is much slower in the second trajectory halves than the first, which suggests that these systems started to settle with respect to the binding of Mg^2+^ ions.

In order to investigate preferred occupation sites of Mg^2+^ ions in the MD simulations independent of their starting positions, an additional MD simulation was set up in which the Mg^2+^ ions were initially placed at least 10 Å from the RNA. As a result, preferred occupation sites between the RNA backbones were observed after 300 ns of MD simulations that are in very good agreement with binding sites of [Co(NH_3_)_6_]^3+^ in crystal structures of Gsw aptamers [8,18] (Fig 1E): Four out of six experimental binding sites in PDB ID 4FE5 were occupied between 25% to 74% of the simulation time of 550 ns; two additional binding sites from PDB ID 3RKF were occupied ~57% of the simulation time (S1 Table). Similar occupation rates were observed in the 18 MD simulations for which the Mg^2+^ ions had been placed at electrostatically favorable positions along the RNA (S1 Table). Likewise, nucleotides that show chemical shift changes upon Mg^2+^ titration to Gsw^loop^ in NMR experiments [18] were found in a distance < 5 Å to the preferred occupation sites of Mg^2+^ ions in our MD simulation (Fig 1E). Note that one cannot expect a perfect agreement of computed occupation sites with experimentally determined ones. On the one hand, this is partly due to the conformational dynamics of the RNA during the MD simulations, which can lead to a shift of an occupation site with respect to the (static) crystal structures. On the other hand, the occupation of sites in the two crystal structures already differs quantitatively (shift between red and orange spheres in Fig 1E) and qualitatively (occupation in one crystal structure but not the other; Fig 1E). Yet, when comparing the occupancies (S1 Table) in terms of ratios of mole fractions of an occupied *versus* unoccupied binding site between different MD trajectories (e.g., for binding site 1, Gsw^apt^ 12 Mg^2+^, and trajectories 1 and 2: (10/90) / (27 / 63)), for the overwhelming majority of the 96 cases (4 systems x 8 binding sites x 3 ratios), this ratio is close to one order of magnitude, and in only four cases it exceeds two orders of magnitude (the 16 cases where the occupancy was either 0 or 100% were not considered here); in our view, this observation displays an encouragingly high internal consistency of occupancies between different trajectories.

In summary, while these results do not prove convergence of the occupation of Mg^2+^ sites, they suggest that the respective systems sufficiently settled, and in a consistent manner, with respect to Mg^2+^ ion binding such that they can be compared to each other for investigating the influence of the concentration of Mg^2+^ ions on the structure and dynamics of Gsw^apt^ and Gsw^loop^.

### The aptamer domain remains globally stable during the MD simulations but shows local structural changes

Data on structural deviations of Gsw^apt^ and Gsw^loop^ are given in Table 1 in terms of mean root mean-square deviations (RMSD) over three trajectories after root mean-square fitting of the conformations on the initial conformation, considering for fitting only those 80% of the nucleotides that show the lowest RMSF (S2 Table). All simulations show mean RMSD ≤ 3 Å for the whole aptamer structures (also exemplarily shown in S3 Fig as a function of simulation time for Gsw^apt^), except for Gsw^loop^ in the absence of Mg^2+^ ions (4.2 Å). RMSD values of up to 3–4 Å have also been observed in other MD simulations of small riboswitches applying a similar simulation setup [42–44]. Accordingly, the comparison of the average conformation over the last 50 ns of MD simulations with the crystal structure shows only small overall structural deviations (S4 Fig). The ff99 force field used here has been reported to lead to irreversible transitions to unexpectedly open and under-twisted RNA structures resembling a ladder due to *anti* to high-*anti* shifts of the χ dihedral [43,45–47]. However, visual inspection of our MD trajectories neither revealed the formation of such ladder-like structures for Gsw^apt^ nor for Gsw^loop^ (S4 Fig). Furthermore, histograms of χ dihedrals using the ff99 force field on Gsw^apt^ and Gsw^loop^ in the presence of 20 Mg^2+^ ions are highly similar to those obtained for the ff10 force field, including the high-*anti* region (S5 Fig for the entire aptamer and S6 Fig for the P1 region only). We also note that some nucleotides in the bound crystal structures of Gsw^apt^ and Gsw^loop^ do occupy the χ high-*anti* region [33].

**Table 1:** Root mean square deviations of Gsw^apt^ and Gsw^loop^ as a whole and for substructures^[a]^.

**Simulated system**		**Aptamer** ^[b]^	**P1**^[c]^	**P2**^[c]^	**P3**^[c]^	**L2**^[c]^	**L3**^[c]^
Gsw^apt^	0 Mg^2+^	3.0 ± 0.1	7.6 ± 0.5	2.4 ± 0.2	2.8 ± 0.2	3.1 ± 0.3	3.9 ± 0.3
	12 Mg^2+^	2.9 ± 0.1	5.3 ± 0.4	2.0 ± 0.1	2.5 ± 0.1	2.1 ± 0.2	3.5 ± 0.2
	20 Mg^2+^	2.3 ± 0.1	4.2 ± 0.3	1.6 ± 0.1	1.7 ± 0.1	2.1 ± 0.1	2.9 ± 0.2
Gsw^loop^	0 Mg^2+^	4.2 ± 0.2	9.9 ± 1.1	3.0 ± 0.2	3.7 ± 0.2	4.3 ± 0.3	5.4 ± 0.4
	12 Mg^2+^	2.4 ± 0.1	4.6 ± 0.5	1.9 ± 0.1	1.9 ± 0.1	3.3 ± 0.2	3.8 ± 0.3
	20 Mg^2+^	2.5 ± 0.1	4.4 ± 0.3	1.8 ± 0.1	2.1 ± 0.1	2.9 ± 0.2	3.6 ± 0.2
**Simulated system**		**J1/2**^[c]^	**J2/3**^[c]^	**J3/1**^[c]^			
Gsw^apt^	0 Mg^2+^	2.3 ± 0.1	3.4 ± 0.2	2.7 ± 0.2			
	12 Mg^2+^	2.4 ± 0.1	4.6 ± 0.2	3.2 ± 0.2			
	20 Mg^2+^	2.0 ± 0.1	3.8 ± 0.3	2.6 ± 0.2			
Gsw^loop^	0 Mg^2+^	3.3 ± 0.3	5.6 ± 0.3	5.2 ± 0.5			
	12 Mg^2+^	1.9 ± 0.1	4.1 ± 0.2	2.3 ± 0.2			
	20 Mg^2+^	2.0 ± 0.1	3.4 ± 0.2	2.8 ± 0.1			

^[a]^ In Å; given is the mean ± SEM calculated over the three trajectories; the first 50 ns of each trajectory were omitted for the calculation. The RMSD was calculated after root mean-square fitting of the conformations on the initial conformation, considering for fitting only those 80% of the nucleotides that show the lowest RMSF (S2 Table).

^[b]^ The complete RNA (nucleotides 15–81).

^[c]^ RMSD of the respective substructure.

Mean RMSD for substructures of Gsw^apt^ and Gsw^loop^ were calculated after root mean-square fitting of those nucleotides showing the 80% smallest RMSF (“core nucleotides”) (Table 1, S2 Table). Together with small RMSD values observed for all substructures but J2/3 when fitting the respective substructure onto itself (S3 Table), these RMSD values thus mostly report on relative motions of the substructure with respect to the core nucleotides. The largest values were observed for the P1 region, up to 6 Å for MD simulations in the presence of Mg^2+^ and 7.6 Å (9.9 Å) for Gsw^apt^ (Gsw^loop^) in the absence of Mg^2+^. Note, however, that these RMSD values result in part from the high mobility of the three terminal base pairs of P1. The P2 and P3 regions show overall the lowest mean RMSD values (≤ 2.8 Å, except for Gsw^loop^ in the absence of Mg^2+^ (3.0–3.7 Å)), as expected for paired regions in the center of the RNA. The larger structural deviations observed in the absence of Mg^2+^ result from relative motions of the P2 and P3 regions with respect to each other due to the lack of charge compensation by the ions. Regarding loops L2 and L3 of Gsw^apt^, a similar trend is observed with mean RMSD values that are higher by about 1 Å in the absence of Mg^2+^ than in its presence; L2 and L3 of Gsw^loop^ show mean RMSD values that are yet higher by 1 Å than those of Gsw^apt^. Finally, of the junction regions, J1/2 shows the lowest mean RMSD (~2 Å) in most simulations; the largest value (3.3 Å) is observed for Gsw^loop^ in the absence of Mg^2+^ ions. In contrast, the J2/3 region shows high mean RMSD values > 3 Å for all simulations as does the J3/1 region (~3 Å). Again, the only exception is Gsw^loop^ in the absence of Mg^2+^ ions where we observe mean RMSD values > 5 Å.

In summary, no gross conformational changes of the aptamer domain were observed during our MD simulations. On a local scale, the mutation in Gsw^loop^ induces local conformational rearrangements in the loop region as well as at the ligand binding site (J1/2, J2/3 and J3/1), manifested in higher RMSD values compared to the simulations of Gsw^apt^.

### The G37A/C61U mutation disturbs the native hydrogen bond network in the loop region

The tertiary interactions between the loops L2 and L3 are formed via a hydrogen bond network composed of two base quadruples. Each quadruple is formed by a canonical Watson-Crick base pair and a non-canonical base pair connected to it. The G37A/C61U mutation is located in the upper base quadruple, which is formed by the Watson-Crick base pair of nucleotides 37 and 61 and the two nucleotides 34 and 65 (Fig 1C). The lower base quadruple is identical in both Gsw variants and consists of the Watson-Crick base pair formed by nucleotides 38 and 60 and the two nucleotides 33 and 66 (S7 Fig).

We calculated average base pair occupancies for native base pairs in the loops L2 and L3 from the occupancies of the hydrogen bonds that are involved in the formation of the base pair (Table 2). For Gsw^apt^ in the absence of Mg^2+^ ions, almost all base pairs, except for A66-G38 from the lower base quadruple, are formed for > 50% of the time. The most stable base pairs are the Watson-Crick base pairs C61-G37 (99%) and C60-G38 (98%), which are also described as the most crucial components of the tertiary interaction [20]. The presence of Mg^2+^ ions in the simulations of Gsw^apt^ results in a stabilization of almost all hydrogen-bonded base pairs and in occupancies for the hydrogen bonds of at least 78% except for the base pair A66-G38 (61%). The occupancies in the presence of Mg^2+^ ions are up to 44% higher than in the absence of Mg^2+^ ions.

**Table 2:** Hydrogen bond occupancy in the L2/L3 loop region of Gsw^apt^ and Gsw^loop^ ^[a]^.

Gsw variant	Nucleotide pair	0 Mg^2+^ ^[b]^	12 Mg^2+^ ^[b]^	20 Mg^2+^ ^[b]^
Gsw^apt^	U34—G37	69.9 ± 6.6	92.8 ± 3.5	78.7 ± 4.8
	U34—A65	68.4 ± 4.2	94.6 ± 1.0	95.8 ± 1.0
	G37—C61	99.1 ± 0.2	99.8 ± 0.1	95.0 ± 2.7
	C61—A65	58.4 ± 7.5	97.1 ± 1.7	93.7 ± 3.9
	A33—A66	52.2 ± 5.8	96.9 ± 0.6	89.5 ± 2.6
	G38—C60	98.0 ± 0.3	96.6 ± 1.1	90.3 ± 3.4
	G38—A66	45.6 ± 5.1	79.2 ± 1.7	61.3 ± 3.2
Gsw^loop^	U34—A37	-^[c]^	-^[c]^	-^[c]^
	U34—A65	54.6 ± 6.5	81.3 ± 3.5	68.7 ± 6.0
	A37—U61	86.4 ± 4.3	76.2 ± 5.3	66.9 ± 5.1
	U61—A65	4.9 ± 4.4	30.6 ± 7.3	24.1 ± 6.8
	A33—A66	28.2 ± 7.6	52.5 ± 5.6	53.6 ± 5.5
	G38—C60	70.1 ± 2.4	99.5 ± 0.1	70.7 ± 2.7
	G38—A66	22.3 ± 4.9	46.0 ± 4.5	64.4 ± 4.5

^[a]^ In %; mean ± SEM over three trajectories of 550 ns length. The nucleotides 34, 37, 61, and 65 belong to the upper base quadruple where the G37A/C61U mutation is located; the nucleotides 33, 38, 60, and 66 form the lower base quadruple. The average value was calculated over the hydrogen bonds connecting the two nucleotides. The SEM was calculated by error propagation.

^[b]^ Number of Mg^2+^ per RNA molecule.

^[c]^ No base pairing for Gsw^loop^.

For Gsw^loop^, the loss of two hydrogen bonds in the upper base quadruple (Fig 1C) results in an overall destabilized hydrogen bond network in our MD simulations, manifested in lower occupancies for the hydrogen bonds in the loop region. In the absence of Mg^2+^ ions, the base pair between the nucleotides 61 and 65 in the upper base quadruple shows hydrogen bond occupancy of only ~5%, i.e., ~50% lower than for Gsw^apt^ in the absence of Mg^2+^. The mutated Watson-Crick base pair A37-U61 yields a value of 86% in the absence of Mg^2+^, ~10% lower than for the G37-C61 base pair in Gsw^apt^. The last base pair in the upper base quadruple (A65-U34) also shows a reduced occupancy in the case of Gsw^loop^ compared to Gsw^apt^. The lower base quadruple is influenced by the mutation as well. Here, the G38-C60 Watson-Crick base pair is again the most stable base pair from this quadruple (70%), whereas the other two base pairs show occupancies of 22% and 28% in the absence of Mg^2+^ ions. As in Gsw^apt^, the presence of Mg^2+^ ions in simulations of Gsw^loop^ results in higher occupancy values for the hydrogen bonds of the hydrogen bond network, except for the interactions between the nucleotides 37 and 61.

In summary, we observe that the hydrogen bond network in the loop region of Gsw^apt^ is more stable than the network in Gsw^loop^, as expected by the introduction of the G37A/C61U mutation in Gsw^loop^. The G37A/C61U mutation does not only decrease the stability of the upper base quadruple in which the mutation is located, but also destabilizes the lower base quadruple. Furthermore, we found that the presence of Mg^2+^ ions results in more stable tertiary interactions for both Gsw^apt^ and Gsw^loop^, in agreement with experimental findings [15,31].

### The presence of Mg^2+^ ions maintains a more compact structure

In order to investigate the influence of Mg^2+^ ions and the G37A/C61U mutation on the compactness of the aptamer domain of Gsw, we calculated the radius of gyration (*R*_g_) of the RNA molecules (Table 3). In the presence of Mg^2+^ ions, the mean *R*_g_ of both Gsw^apt^ (16.2 ± 0.3 Å) and Gsw^loop^ (16.4 ± 0.4 Å) agree with the value calculated for the ligand-bound crystal structures (16.1 Å and 16.3 Å, respectively), which contain [Co(NH_3_)_6_]^3+^ ions in addition. Only minor differences were observed when comparing the mean *R*_g_ from MD simulations with 12 and 20 Mg^2+^ ions. The mean *R*_g_ in the absence of Mg^2+^ ions is increased by ~1 Å for both Gsw^apt^ and Gsw^loop^. The more compact structure in the presence of Mg^2+^ ions is maintained due to the ions compensating the negative charges of the RNA backbone. For all three Mg^2+^ ion concentrations, the differences in mean *R*_g_ between Gsw^apt^ and Gsw^loop^ are only marginal (≤ 0.2 Å), indicating only a minor influence of the mutation on the compactness of the structure.

**Table 3:** Radius of gyration of Gsw^apt^ and Gsw^loop^ ^[a]^.

Gsw variant	0 Mg^2+^ ^[b]^	12 Mg^2+^ ^[b]^	20 Mg^2+^ ^[b]^	Crystal structure ^[c]^
Gsw^apt^	17.2 ± 0.1	16.3 ± 0.1	16.2 ± 0.1	16.1 ^[d]^
Gsw^loop^	17.4 ± 0.1	16.4 ± 0.1	16.4 ± 0.1	16.3 ^[e]^

^[a]^ In Å; given is the mean ± SEM calculated over three trajectories each. The radius of gyration was calculated omitting the P1 region.

^[b]^ Number of Mg^2+^ ions per RNA molecule.

^[c]^ The crystal structures are ligand-bound.

^[d]^ Calculated for PDB ID 4FE5 [17]

^[e]^ Calculated for PDB ID 3RKF [18]

In summary, we observe that the G37A/C61U mutation does not have an influence on the structural compactness of the RNA. The absence of Mg^2+^ ions in the MD simulations results in a less compact structure than when Mg^2+^ is present and in the ligand-bound crystal structures.

### The G37A/C61U mutation increases, whereas Mg^2+^ ions decrease the structural dynamics of the aptamer

**Fig 2:**
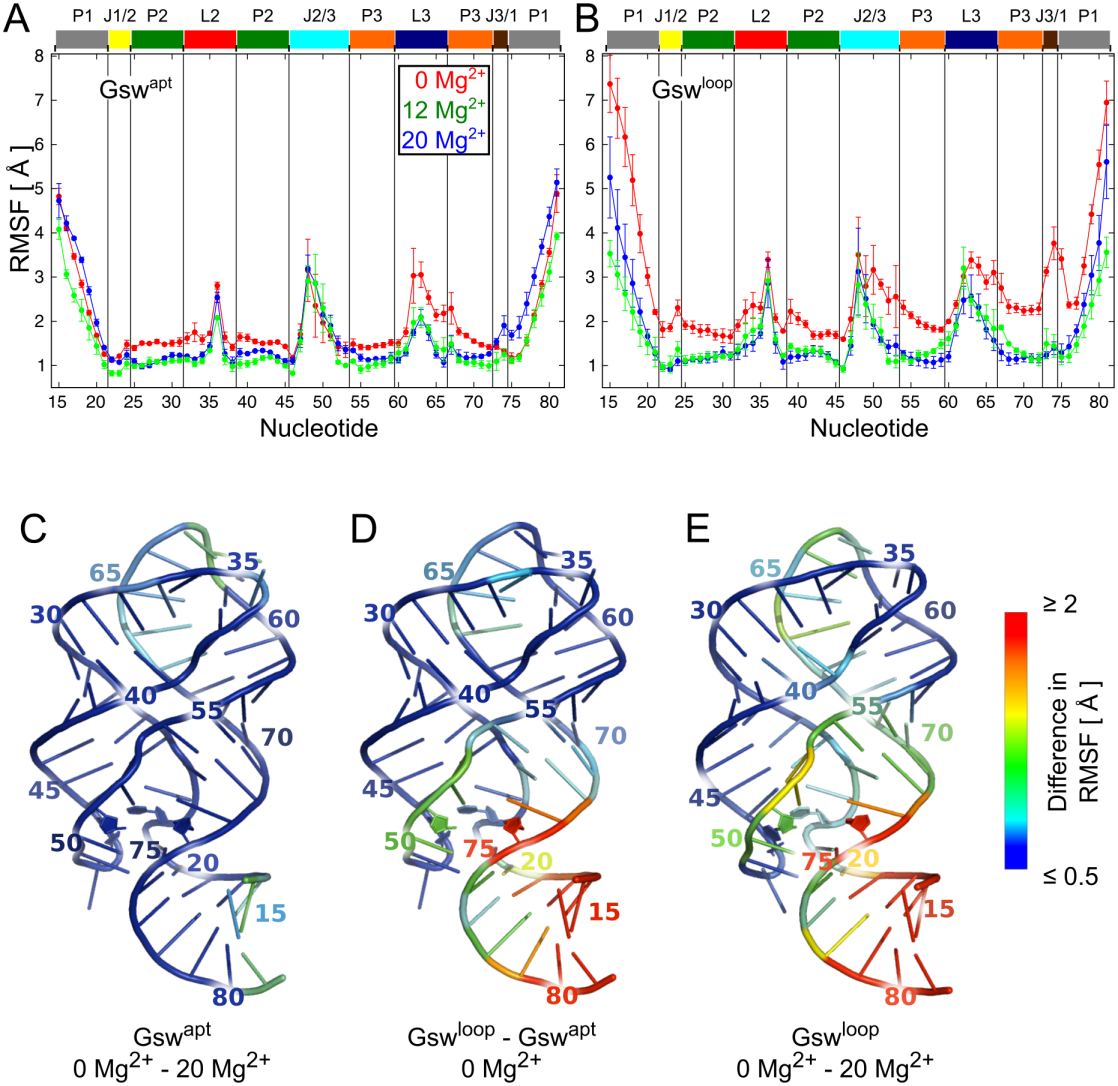
Atomic fluctuations calculated from MD simulations. A, B: Mean (± SEM) atomic fluctuations (RMSF) on a per nucleotide level for Gsw^apt^ (A) and Gsw^loop^ (B) over the three simulations for each system setup. Secondary structure regions are depicted above the plots and colored according to Fig 1A. Red: simulations in the absence of Mg^2+^ ions; green: simulations with 12 Mg^2+^ ions per RNA molecule; blue: simulations with 20 Mg^2+^ ions per RNA molecule. C, D, E: Differences in atomic fluctuations projected onto the RNA structure. Larger differences are colored red, smaller differences blue. Nucleotides responsible for ligand binding are shown as sticks. C: Difference in atomic fluctuations for Gsw^apt^ of 0 Mg^2+^—20 Mg^2+^ ions per RNA molecule. D: Difference in atomic fluctuations in the absence of Mg^2+^ for Gsw^loop^—Gsw^apt^. E: Difference in atomic fluctuations for Gsw^loop^ of 0 Mg^2+^—20 Mg^2+^ ions per RNA molecule.

In order to gain insights into the influence of Mg^2+^ ions and the G37A/C61U mutation on the dynamics of the RNA, we calculated atomic root mean-square fluctuations (RMSF) and averaged them per nucleotide (Fig 2A and 2B). For all simulations, the stem regions P2 and P3 are the least mobile, while the terminal P1 region shows high RMSF. The low mobility of the P2 and P3 regions is in line with SHAPE experiments of the unbound state of a purine riboswitch characterizing these regions among the most stable ones [16].

In all MD simulations, the J2/3 region shows a pronounced mobility in agreement with experiments [16] and the assumption that the J2/3 region acts as an entry gate to the ligand binding site [9]. The fluctuations of nucleotides in the J1/2 and J3/1 regions are similar in magnitude to those in the P2 and P3 regions in all MD simulations, except for Gsw^loop^ in the absence of Mg^2+^ ions. Here, nucleotide 74 in the J3/1 region shows RMSF values that are ~2 Å higher than those of the other simulations. Pronounced fluctuations can also be observed in the L2 and L3 regions. In general, Gsw^loop^ shows a higher mobility than Gsw^apt^, as seen from mean RMSF values calculated over the 80% least fluctuating nucleotides (S2 Table) that are up to ~0.6 Å higher in the former case (S4 Table). The presence of 20 Mg^2+^ ions results in mean RMSF values that are lower by ~0.5 Å (~0.8 Å) for Gsw^apt^ (Gsw^loop^) than in the absence of Mg^2+^ ions.

In Fig 2C, 2D and 2E, differences in mean RMSF on a per-nucleotide level are projected onto the aptamer structure. Comparing the mean RMSF of Gsw^apt^ in the absence and presence of 20 Mg^2+^ ions (Fig 2C), we observe differences ≤ 1 Å at the ends of the terminal P1 region and in the loop region, which points to a subtle Mg^2+^-induced decrease in structural dynamics of the loop region and the adjacent P3 region. Addressing the influence of the G37A/C61U mutation on the aptamer mobility surprisingly reveals that the largest differences in RMSF between Gsw^loop^ and Gsw^apt^ in the absence of Mg^2+^ ions (Fig 2D) are not found at the mutation site in the loop region, but at the ligand binding site ~30 Å away and at the even farther end of the terminal P1 region. At the ligand binding site, particularly J2/3 and J3/1 show higher mobility in Gsw^loop^, with the largest difference calculated for nucleotide 74 (2.4 Å). These results provide the first evidence at the atomistic level that the G37A/C61U mutation has a long range effect in terms of increasing the structural dynamics at the ligand binding site, as has been inferred from the influence on the ligand binding ability of Gsw^loop^ [15,18], *and* the P1 region, which has not been considered so far.

As for Gsw^apt^ but much more marked, we observe a Mg^2+^-induced decrease in structural dynamics of the aptamer for Gsw^loop^: The Mg^2+^ ions now decrease the structural dynamics across the entire aptamer domain (Fig 2E). Large differences (> 2 Å) are found for the ends of the terminal P1 region (between 2.2 and 4 Å) and for the nucleotides in J3/1. Especially nucleotide 74 shows a large decrease in mobility in the presence of Mg^2+^ ions (2.2 Å). In addition, the L3 loop, the P3 region, and the joining J2/3 region are affected by the Mg^2+^-induced decrease in structural dynamics. Finally, the differences in RMSF between Gsw^loop^ and Gsw^apt^ in the presence of 20 Mg^2+^ ions (S8 Fig) are mostly small, with the largest difference (1.2 Å) found for nucleotide 63, which is part of the L3 loop, and other differences (< 1 Å) observed in the loop region.

In summary, we find that the G37A/C61U mutation results in increased structural dynamics of the aptamer domain of Gsw^loop^ compared to Gsw^apt^. This is not only the case in the loop region where the mutation in Gsw^loop^ is located, but interestingly also at the distant ligand binding site *and* the P1 region. Mg^2+^ ions decrease the structural dynamics of the aptamer domains of both Gsw^apt^ and Gsw^loop^. The Mg^2+^-induced decrease of the structural dynamics is much more pronounced in the case of Gsw^loop^, however, such that Gsw^apt^ and Gsw^loop^ behave similarly in terms of atomic mobility in the presence of 20 Mg^2+^ per RNA.

### Particularly in the presence of Mg^2+^, information on ligand binding is channeled to the P1 region

In order to gain further insights into the interplay of the L2-L3 region, the ligand binding core, and the P1 region as a function of ligand binding, we applied a graph-based rigidity analysis as implemented in the FIRST software [48]. The approach converts structural information of an input structure into a constraint network, where vertices represent atoms and edges (constraints) represent covalent and noncovalent interactions (hydrogen bonds, salt bridges, and hydrophobic interactions). Subsequently, the pebble game algorithm [49,50] is applied on the constraint network, which results in a decomposition into rigid regions and flexible links in between. Rigidity analyses have been successfully applied to investigate static properties (flexibility and rigidity characteristics) of proteins [48,51–54], also including analyses on the effect of ligand binding [52,55]. As to RNA, the approach was applied to the large ribosomal subunit [56–58] as well as smaller RNAs [59]. To do so, an RNA-specific parameterization of the constraint network has been developed [59], which is applied here. As suggested earlier [52,60,61], we applied the rigidity analysis to an ensemble of structures in order to improve the robustness of the results.

**Fig 3:**
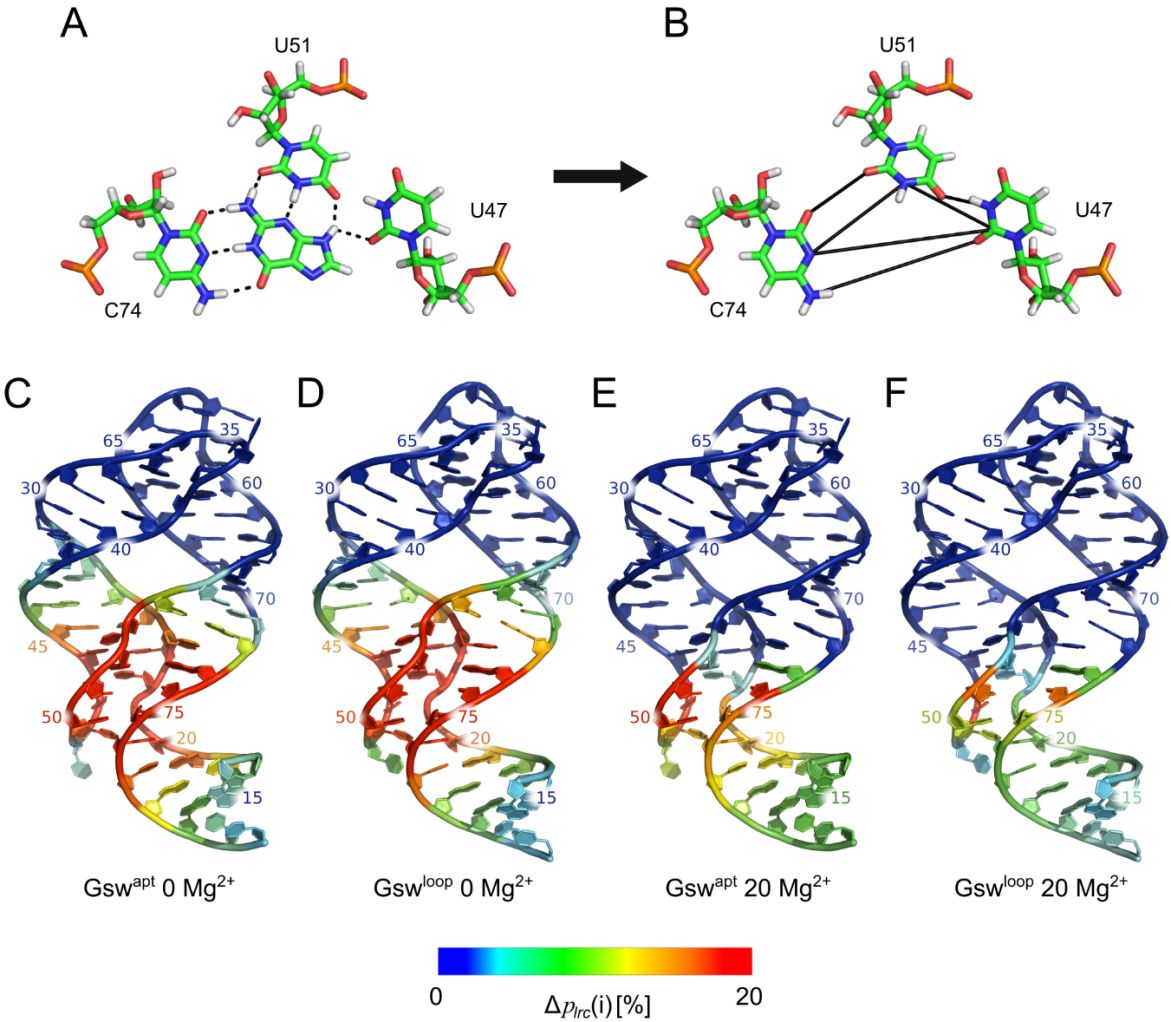
Rigidity analyses. A: Nucleotides involved in binding guanine in the binding site of the aptamer domain of Gsw (PDB ID 1Y27 [26]). Black dashed lines indicate hydrogen bonds. B: Constraints (black lines) added to conformations of the *apo* aptamer domain of Gsw to model the presence of guanine in the binding site for rigidity analyses. C, D, E, F: Nucleotide-wise differences in the probability to be in the largest rigid cluster (Δ***p***_***lrc***_(***i***), Eq 1, ***E***_***HB***_ = **−0.6 kcal/mol** for the rigidity analyses) between the ligand being present or absent in the aptamer domain, projected onto the aptamer domain of Gsw^apt^ (C, E) and Gsw^loop^ (D, F) in the absence of Mg^2+^ ions (C, D) and in the presence of 20 Mg^2+^ ions (E, F).

From the rigid cluster decomposition, we calculated the probability for each nucleotide to be in the largest rigid cluster (*p*_*lrc*_(*i*), Eq 1), as done previously [52], and averaged these results over the MD-derived ensembles of Gsw^apt^ and Gsw^loop^ *apo* structures. In order to simulate the presence of guanine in the binding site of Gsw, we added additional constraints to the network connecting the nucleotides U47, C74, and U51, which bind guanine in the crystal structure (Fig 3A and 3B). According to ref. [59], the number and distribution of constraints will result in the nucleobases of U47, C74, and U51 forming one rigid unit. To probe how this local rigidification will percolate through the aptamer domain, we also calculated *p*_*lrc*_(*i*) per nucleotide in the presence of the ligand constraints and, finally, the difference Δ*p*_*lrc*_(*i*) = *p*_*lrc*_(*i*)_*lig*_ − *p*_*lrc*_(*i*)_*apo*_ (S9 Fig, Fig 3C, 3D, 3E and 3F). The results yield insights into the influence of ligand binding on the location of the largest rigid cluster in the aptamer domain. Note that by construction, any influence due to conformational changes of the RNA between *apo* and ligand-bound state is excluded.

In the absence of Mg^2+^ ions in the MD simulations (Fig 3C and 3D), the presence of the ligand constraints results in an increase of *p*_*lrc*_(*i*) for nucleotides in the J1/2, J2/3 and J3/1 regions, which is not surprising. Similarly, nucleotides in P1, P2, and P3 adjacent to the ligand binding site show an increased *p*_*lrc*_(*i*) in the presence of the ligand constraints, including nucleotides in the “P2 tune box” [17]. These results reveal a stabilization of the ligand binding site and its environment “above and below” due to the presence of ligand constraints.

In the presence of 20 Mg^2+^ ions (Fig 3E and 3F), we observe that the presence of the ligand constraints has only little effect on *p*_*lrc*_(*i*) of nucleotides in the P2, P3, L2, and L3 regions (|Δ*p*_*lrc*_ (*i*)|≤1%). In contrast, the presence of the ligand constraints result in a much larger increase in *p*_*lrc*_(*i*) for nucleotides in the J2/3 region (up to 42%) and in the J3/1 region (up to 20%). As for the absence of Mg^2+^ ions, we again observe an increase in *p*_*lrc*_(*i*) for all nucleotides in the P1 region by the presence of the ligand constraints, even though the values are higher in the absence of Mg^2+^ ions. Thus, in the presence of 20 Mg^2+^ ions, the stabilizing effect of ligand constraints is more local and channeled towards the P1 region.

The detailed comparison of Δ*p*_*lrc*_(*i*) between Gsw^apt^ and Gsw^loop^ in the absence of Mg^2+^ ions (Fig 3C and 3D) shows that the influence of the ligand constraints is similar by and large for the two variants, although differences up to ~5% in Δ*p*_*lrc*_(*i*) are found. Nucleotides in the binding region show a stronger influence of the ligand constraints in Gsw^loop^. This is likely due to the larger flexibility observed for Gsw^loop^ in the binding region in the *apo* state, which becomes more restricted by the presence of the ligand constraints. In contrast, the presence of the ligand constraints has a slightly larger influence on nucleotides in the P1 region for Gsw^apt^ (up to 3%) than for Gsw^loop^. In the presence of 20 Mg^2+^ ions, the stabilizing effect of the ligand constraints towards the terminal ends in the P1 region is more pronounced for Gsw^apt^, which shows Δ*p*_*lrc*_(*i*) values for this region 2 to 7% higher than for Gsw^loop^, with the higher values being closer to the ligand binding site.

In summary, we observe an increase in *p*_*lrc*_(*i*) in the presence of ligand constraints for nucleotides at the ligand binding site and in its environment. In Gsw^apt^ and when 20 Mg^2+^ ions are present, the rigidifying effect towards the terminal ends of P1 is largest. Considering that the tertiary loop interactions are already preformed in the unbound state of Gsw^apt^ even in the absence of Mg^2+^ ions [31,32], yet are further strengthened in the presence of Mg^2+^ [31,62], this finding suggests that both the L2-L3 region and ligand binding cooperatively stabilize the P1 region.

### Tertiary interactions in the loop region and ligand interactions cooperatively stabilize the terminal nucleotides in the P1 region

In order to verify that the tertiary loop-loop interactions and the presence of the ligand constraints rigidify the terminal nucleotides in the P1 region cooperatively, we computed the cooperative effect *Coop*(*i*) on the stabilization of nucleotides *i* in the P1 region from respective *p*_*lrc*_(*i*) values according to Eq 2. If *Coop*(*i*) > 0, interactions at both sites together have a larger effect on nucleotide *i* than the sum of the effects of the separate interactions.

**Fig 4:**
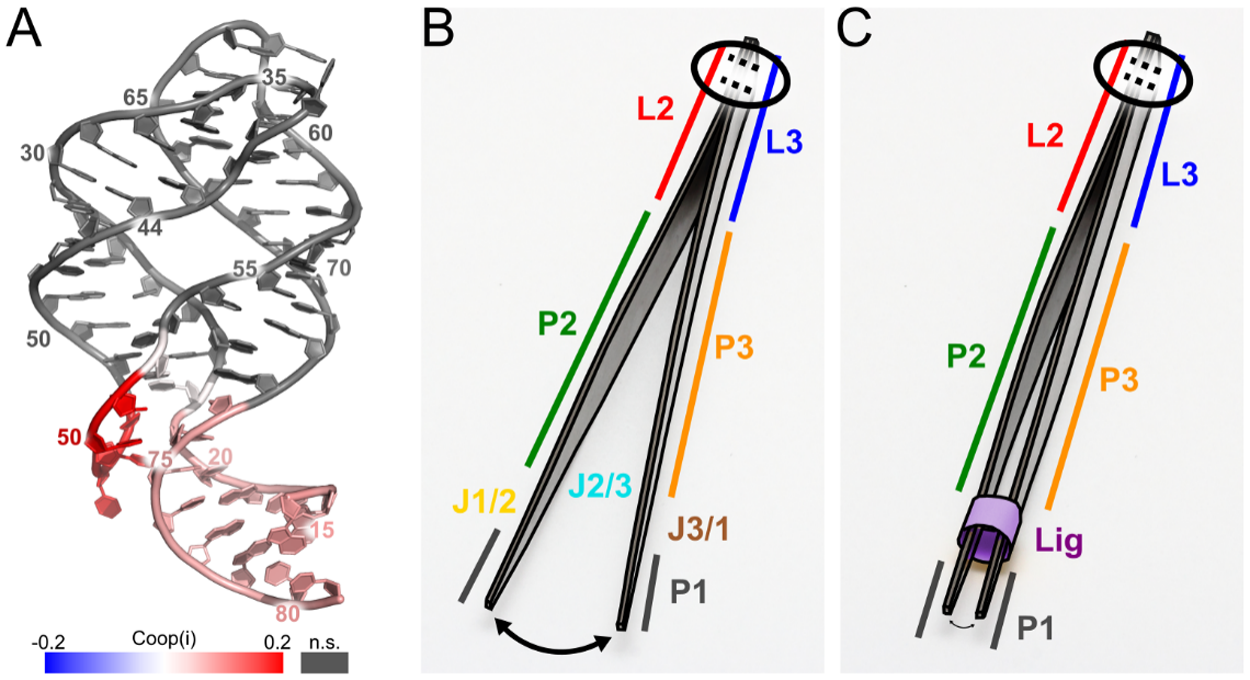
Cooperative influence on the P1 region. A: *Coop*(*i*) values mapped onto the aptamer. The values were calculated according to Eq 2 for the systems in the presence of 20 Mg^2+^ ions (see also Table 4). Grey nucleotides show *Coop*(*i*) values that are not significantly different from zero (*p* > 0.05). B, C: Model for the influence of tertiary interactions and ligand binding on the stability of the P1 region. The tweezer represents the aptamer domain of the guanine-sensing riboswitch. The secondary structure elements are indicated by colors as in Fig 1A. The tertiary interactions are shown as dotted lines at the top of the tweezers and are encircled. The flexibility of the P1 region is indicated by the differently sized arrows at the bottom. B: The ligand-unbound state; C: The ligand (purple) has a stabilizing influence on the P1 region, but more so if the tertiary interactions are present.

For the terminal nucleotides in the absence of Mg^2+^ ions, almost all of the nucleotides in the five terminal base pairs show *Coop*(*i*) values that are not significantly different from zero (Table 4), demonstrating that in this case the L2-L3 region and the ligand binding do not act cooperatively on P1. The only exception is nucleotide 77, which is close to the ligand binding site. In contrast, in the presence of 20 Mg^2+^ ions, all nucleotides in the five terminal base pairs show *Coop*(*i*) values that are positive and significantly or even highly significantly different from zero (Table 4, Fig 4A), demonstrating that with a structurally more stable aptamer domain, the L2-L3 region and the ligand binding act cooperatively on P1.

**Table 4:** *Coop*(*i*) values for the terminal nucleotides in the P1 region^[a]^.

Nucleotide numbers	0 Mg^2+^		20 Mg^2+^	
	*Coop*(*i*) ± SEM ^[b]^	*p* ^[c]^	*Coop*(*i*) ± SEM ^[b]^	*p* ^[c]^
15	0.009 ± 0.038	ns	0.065 ± 0.021	**
16	0.003 ± 0.036	ns	0.060 ± 0.019	**
17	-0.020 ± 0.036	ns	0.061 ± 0.017	***
18	-0.043 ± 0.037	ns	0.060 ± 0.016	***
19	-0.090 ± 0.040	ns	0.062 ± 0.015	***
77	-0.136 ± 0.039	**	0.062 ± 0.015	***
78	-0.043 ± 0.037	ns	0.060 ± 0.016	***
79	-0.020 ± 0.036	ns	0.061 ± 0.017	***
80	0.001 ± 0.036	ns	0.060 ± 0.019	**
81	0.005 ± 0.039	ns	0.068 ± 0.021	**

^[a]^ *Coop*(*i*) values were calculated based on Eq 2.

^[b]^ The SEM was calculated via error propagation from the single terms of Eq 2.

^[c]^ The *p* value was calculated using a two-sided one-sample *t*-test and the null hypothesis of equality to zero.

* *p* < 0.05;

** *p* < 0.01;

*** *p* < 0.001;

ns: *p* ≥ 0.05

In summary, the stabilization of the L2-L3 region by tertiary interactions and the ligand binding site by ligand binding cooperatively influences the structural stability of the terminal base pairs in the P1 region in the presence of Mg^2+^ ions.

## Discussion

We investigated the interplay of structural stability of the L2-L3 region, the ligand binding core, and the P1 region in the aptamer domain of Gsw by MD simulations and rigidity analyses of the wildtype structure and the G37A/C61U mutant. As the main result, we found that stabilization of the L2-L3 region by tertiary interactions, and the ligand binding site by ligand binding, cooperatively influences the structural stability of the terminal base pairs in the P1 region in the presence of Mg^2+^ ions.

Previous, comprehensive studies had revealed intricate relationships between ligand binding to the aptamer domain of Gsw and presence of tertiary L2-L3 interactions [8,19] [16], as well as between ligand binding and influence on the P1 region [8] [16]. These studies stressed that subtle (structural) changes, such as the loss of two hydrogen bonds in the Watson-Crick base pair in the upper base quadruple in the L2-L3 region or the presence or absence of Mg^2+^ ions, profoundly affect the function of the aptamer domain, indicating that the overall structural stability of this domain in the absence of a ligand is marginal. While yielding valuable data to validate our simulation and modeling data with, these findings also provide a challenge in terms of appropriately parameterizing and setting up our computations.

As to the parameterization, contemporary force fields for RNA usually well describe canonical helices; however, they may show different successes on more complex RNAs [63–65]. To investigate to what extent a force field can bias insights gained from MD simulations, we had performed MD simulations with an aggregate simulation time of > 11 μs in previous work [33], probing the influence of the Amber ff99 force field [66] as well as of ff99 with parmbsc0 [67] and parmχOL [68] modifications on the aptamer’s properties. There, ff99 yielded the best agreement with experimental observations, which provided the incentive for us to use ff99 also in the present study. Furthermore, results of rigidity analyses depend on the definition when to include a constraint into the network representation to model a noncovalent interaction [48]. In previous work, we had parameterized a constraint network representation for RNA structures [59]. The modifications were verified by comparing predictions from rigidity analysis to mobility information derived from crystallographic B-values and by predicting and comparing conformational variabilities of RNA structures to those of NMR ensembles [59]. This constraint network representation was used here. Finally, modeling Mg^2+^ ions in MD simulations is considered challenging [36,37,39,64]. Following our earlier findings [33], we used the Mg^2+^ parameters developed by Åqvist [69] and placed them by a two-step procedure including the first hydration shell. This treatment resulted in a pronounced mobility of the Mg^2+^ ions during the first ~100 ns of the MD simulations, suggesting sufficient time for them to equilibrate around the aptamer domain. As a result, preferred occupation sites of the Mg^2+^ ions agree very well with experimentally determined ones from crystal structures and NMR experiments and with respect to different MD trajectories for one system. It is not surprising, though, that we do not obtain perfect agreement with the experimentally determined Mg^2+^ ion binding sites, as even two crystal structures do not yield exactly identical occupation of the binding sites (Fig 1E). Therefore, our setup and modeling of Mg^2+^ ions led to results that suggest that the respective systems sufficiently settled, and in a consistent manner, with respect to Mg^2+^ ion binding such that they can be compared to each other for investigating the Mg^2+^ dependence on the structure and dynamics of Gsw^apt^ and Gsw^loop^.

Likely the largest challenge arose from modeling the structural dynamics of Gsw^apt^ and Gsw^loop^ in the *apo* state, starting from the conformations of the ligand-bound state extracted from crystal structures [8,18]. This challenge is reflected in that even for a less complex B-DNA structure, already ~5 μs of simulation time was required to achieve converged results (63). At present, no experimental structural information at the atomistic level of the unbound aptamer domain of Gsw is available. Yet, the facts that the aptamer domain of Gsw is one of the smallest riboswitch aptamer domains structurally known and that the global fold of the aptamer domain is already present in the unbound state [11,18,19,31,32] make the Gsw aptamer domain a seemingly well-suited system for our investigations. Experimental findings suggest that no large-scale conformational changes need to be simulated to reach the *apo* state [70]. Accordingly, the structural ensembles generated in our MD simulations display pronounced structural characteristics in very good agreement with experiment. At the global level, we found that the mean *R*_g_ of both Gsw^apt^ and Gsw^loop^ in the presence of 20 Mg^2+^ ions agree with respect to absolute and relative magnitudes with values calculated for the respective crystal structures (Table 3), despite local structural rearrangements in the loop region and at the ligand binding site induced by the double mutation in Gsw^loop^ (Table 1). Furthermore, we found that the mean *R*_g_ in the presence of Mg^2+^ ions is significantly (*p* < 0.005) reduced for both Gsw^apt^ and Gsw^loop^, in agreement with experimental results on other riboswitch aptamer domains [71].

The tertiary interactions in the L2-L3 region play an important role for the structural stability and the ligand binding ability of the aptamer domain [8,16]. In experiments, the introduction of the G37A/C61U mutation caused a destabilization of the tertiary interactions and thereby influenced the ligand binding ability of Gsw^loop^ in a Mg^2+^-dependent manner [31]. In our MD simulations, we did not observe a complete disruption of the tertiary interactions in Gsw^loop^ in the absence of Mg^2+^ ions, in contrast to NMR experiments where no tertiary interactions were found [18]; yet, hydrogen bond occupancies in the upper base quadruple reduced by up to 50% compared to Gsw^apt^ were found, as were reduced occupancies in the lower base quadruple (Table 2). Thus, while the first finding indicates that our simulation length is likely not long enough to reach an exact structural state of the loop L2-L3 region of *apo* Gsw^loop^ when starting from the crystal structure with formed tertiary interactions [18], the second finding suggests that our structural ensembles do reflect important differences between Gsw^apt^ and Gsw^loop^. Furthermore, we observe a stabilization of the hydrogen bond network in the presence of Mg^2+^ ions, for both Gsw^apt^ and Gsw^loop^, in agreement with experimental findings [15]. Hence, the ability to model the destabilized hydrogen bond network in the loop region in the case of Gsw^loop^ allows us to use Gsw^loop^ as a test system for investigating the influence of the absence of stable tertiary interactions on the structure and dynamics of the aptamer domain.

In general, we find that Gsw^loop^ shows higher atomic fluctuations than Gsw^apt^, especially in the absence of Mg^2+^ ions (Fig 2A, 2B and 2D; S4 Table). The presence of Mg^2+^ ions results in a smaller difference in atomic fluctuations between Gsw^apt^ and Gsw^loop^, reducing the effect of increased structural dynamics due to the mutation in Gsw^loop^ (Fig 2A and 2B; S8 Fig; S4 Table). In more detail, the largest mobility for all simulated systems occurs in the J2/3 region. This is in agreement with the fact that the J2/3 region was experimentally found to be the most flexible region in the unbound aptamer [16], and is in accordance with the assumption that J2/3 acts as an entry gate to the binding site [9]. Furthermore, our MD simulations show that the junction region shows decreased structural dynamics in the presence of Mg^2+^ ions compared to their absence, especially in Gsw^loop^ for which the stability of the tertiary interactions is dependent on Mg^2+^ ions as well. This is in agreement with results from chemical probing experiments that showed that the majority of the binding site experiences a significant reduction in conformational freedom upon formation of loop-loop interactions [16]. The J3/1 junction is another region that is influenced by the presence or absence of the tertiary interactions (Table 1, Fig 2). This region remained generally stable in our MD simulations, except for the simulations of Gsw^loop^ in the absence of Mg^2+^ ions, as seen from high RMSD and RMSF values in this case. The high mobility in the absence of Mg^2+^ ions in connection with the decreased mobility in the presence of Mg^2+^ ions could explain the experimental observation that Gsw^loop^ is not able to bind hypoxanthine in the absence of Mg^2+^ ions, while the hypoxanthine binding ability is restored in the presence of Mg^2+^ ions [18]: Nucleotide C74, located in J3/1 and hypothesized to be involved in the initial binding of the ligand [9,16], might exhibit too little stability for productive binding of the ligand in the absence of Mg^2+^. This would be in line with, and extend, the atomistic understanding of the hypothesis that the formation of the initial encounter complex relies on a restricted conformational dynamics and a partial structural organization of the ligand binding core region [15]. The conformational restriction and partial organization of the ligand binding core has been hypothesized to be induced through stabilization of remote regions [15,16]. Indeed, we observed that the presence of Mg^2+^ ions in simulations of Gsw^loop^ results in a more stable hydrogen bond network in the loop region and a related stabilization of the ligand binding site.

In addition to the influence on the ligand binding core, our analyses of RMSF in the absence of Mg^2+^ reveal that the G37A/C61U mutation also increases the structural dynamics in the P1 region, which, to the best of our knowledge, has not been described before (Fig 2). The P1 region is about 40–50 Å away from the L2-L3 region and contains part of the switching sequence. The presence of Mg^2+^ reduces the structural dynamics particularly in that region, as indicated by a reduction in RMSF of > 2 Å (Fig 2), and this effect is stronger in Gsw^loop^ than in Gsw^apt^. Together with the findings that the presence of Mg^2+^ restores the tertiary interactions in the L2-L3 region in Gsw^loop^ (Table 2), and further strengthens them in Gsw^apt^ [15] (Table 2), these results provide another indication that the tertiary interactions in the L2-L3 region exert a stabilizing effect on the P1 region.

The information about ligand binding and its effect has been shown or inferred to be transmitted to both the L2-L3 and P1 regions [8,14,16,21–23]. Our finding that the presence of the tertiary interactions in the L2-L3 region is transmitted to the P1 region then leads to the hypothesis that these three regions do not only “communicate” in a pairwise manner but that there is a cooperative interplay among all three. The cooperativity likely, at least in part, originates from dynamic allostery [72], i.e. due to changes in the structural dynamics upon ligand binding rather than by macromolecular conformational changes. To probe this interplay, we performed graph theory-based rigidity analyses [48,49] on the MD-derived structural ensembles of Gsw^apt^ and Gsw^loop^ in the *apo* state, modeling the presence of a ligand in the binding site by adding constraints to the network representation of the aptamer domains. In contrast to comparing the influence of a ligand in “true” *holo* ensembles of Gsw^apt^ and Gsw^loop^, our procedure has the advantage that only the influence of additional interactions due to the ligand are detected, whereas potential ligand-induced conformational changes of the aptamer domain are excluded by definition. Obviously, the result of our ligand modeling will depend on the strength with which we couple the ligand to the aptamer network. It is important to note that we used a “weak” coupling scheme as we only provide constraints for hydrogen bonds that a ligand would form with and introduce between the three nucleobases U47, U52, and C47, according to information from crystal structures [8,26]. In particular, we do not include additional constraints for base stacking interactions between the ligand and nucleotides of the P1 region, nor those between the ligand and the base triple located above [8,26]. Such interactions would only increase effects due to ligand binding on the L2-L3 and/or P1 regions.

As a direct measure of the ligand influence on the structural stability, we evaluated the nucleotide-wise probability of finding a nucleotide in the largest rigid cluster across the structural ensemble, *p*_*lrc*_(*i*). This measure had been used successfully to analyze stabilizing effects on the binding partners in the context of protein-protein complex formation [52]. Notably, while in all cases an increase in *p*_*lrc*_(*i*) in the presence of ligand constraints was found for nucleotides at the ligand binding site and in its environment, the rigidifying effect towards the terminal ends of P1 was largest for Gsw^apt^ and when 20 Mg^2+^ ions were present (Fig 3). Computation of the cooperative effect *Coop*(*i*) then confirmed that stabilization of the L2-L3 region by tertiary interactions, and the ligand binding site by ligand binding, cooperatively influences the structural stability of the terminal base pairs in the P1 region in the presence of Mg^2+^ ions (Table 4). Hence, our computations reveal for the first time that there is a structurally stabilizing interplay among the L2-L3 region, the ligand binding core, and the P1 region in the aptamer domain of Gsw^apt^. Accordingly, both the L2-L3 region and the ligand binding contribute to a more stable terminal part of the P1 region, the consequence of which could be that the adjacent switching region is not available for the formation of the antiterminator and, therefore, the transcription terminator loop can form [8,16]. A simple mechanical analog to this allosteric mechanism are tweezers (Fig 4B and 4C), where the mechanically connected end corresponds to the tertiary interactions, which are inherently necessary for the functional stability. The variability in the terminal ends of the tweezers, equivalent to P1, can be restrained by a sleeve (purple in Fig 4C) corresponding to the ligand at the ligand binding site. (Note that this analogy does not take into account that the ligand also has a stabilizing effect on the L2/L3 region.) Our functional insights may provide an explanation as to why the three-way junction is a central structural element also in other purine riboswitches and in the TPP riboswitch [12].

As to the role of Mg^2+^, the Mg^2+^ concentration in *Bacillus subtilis* cells is estimated to be in the millimolar range [73], similar to the intracellular Mg^2+^ concentrations in the range of ~0.5 to 2 mM reported for other bacteria [74]. For intracellular riboswitch concentration, values between 1 to 100 nM have been estimated [75,76]. Together, this would result in [Mg^2+^]:[RNA] ratios that embrace the critical [Mg^2+^]:[RNA] ratio of ~20, above which the rigidifying effect towards the terminal ends of P1 is largest. Therefore, the cooperative effect detected here could become contextual [77] in that the same system of molecular components, Gsw and its ligands, can display different properties depending on the [Mg^2+^]:[RNA] ratio.

Regarding the significance of our findings for the full aptamer’s switching function, by definition any conformational changes of the aptamer domain were excluded upon modeling the presence of the ligand for the rigidity analyses. In the absence of a conformational change, the observed stability changes relate to changes in the width of the states [78], such that the transmission of allosteric information from the ligand binding core to the P1 region can be entropy-dominated, as suggested by Cooper and Dryden [72] and Tsai *et al*. [79]. This result emphasizes the role of changes in the aptamer’s dynamics for the allosteric communication [72]. Further confirmation for this entropy-dominated allosteric communication could be obtained from rigorous entropy calculations [80], which are beyond the scope of this study, however, or from NMR relaxation studies of dynamics in RNA. The proposed allosteric mechanism is in line with models that favor a high degree of preorganization of the unbound state of the aptamer domain [31,32,62], because according to this mechanism no large structural changes are required to transmit information about a bound ligand to the terminal base pairs of the P1 region. Our finding would also be supported by the fact that the ligand binding time (~0.04–0.25 s as estimated from on-rates for guanine binding of 0.2–1.5 * 10^5^ M^-1^ s^-1^ for Gsw variants [9,17] and a guanine concentration of ~190 μM in *E*. *coli* [81]) is already comparable to the time required to transcribe the full aptamer (considering a transcription rate of the RNA polymerase of ~50 nt/s [17] and a length of the expression platform of ~100 nucleotides [8,82]) such that a quick stabilization of the P1 region is required in order to drive the formation of the transcription terminator in time for the subsequent regulation [83].

In our study, we only investigated the aptamer domain of the two Gsw variants Gsw^apt^ and Gsw^loop^. A recent NMR study of the structurally similar adenine-sensing riboswitch uncovered a three-state behavior of the complete riboswitch including the expression platform [75]: A ligand bound state, a similar ligand-unbound yet binding-competent state, and a newly found ligand-unbound state incapable of ligand binding. Thus, ideally further studies should include the expression platform as well. However, due to the scarcity of structural information for the expression platform and the long time-scales for folding of RNA structures compared to the time-scales of standard MD simulations, the inclusion of the expression platform in all-atom MD simulations is difficult.

## Methods

### Molecular dynamics simulations

The setup of the molecular dynamics (MD) simulations in the present study is similar to that in our previous work [33]. However, the MD simulations performed here are 2.5 times longer than before. In detail: MD simulations were performed with the Amber 11 suite of programs [84,85] and the force field ff99 [86]. The starting structures for the MD simulations were obtained from ligand bound crystal structures. The ligands were removed from these, and the sequence of the wild type structure (PDB ID: 4FE5 [17]) was adapted to match the sequence of the G37A/C61U mutant (PDB ID: 3RKF [18]). For this, the coordinates of the bases of the mutant were copied after superimposing the wild type structure. As a result, wild type and mutant only differed in nucleotides 37 and 61. The modified wild type will be referred to as Gsw^apt^, and the G37A/C61U mutant will be referred to as Gsw^loop^, as introduced by Schwalbe *et al*. [18]. For both structures, three simulation systems with different Mg^2+^ concentrations (0, 12 and 20 Mg^2+^ ions per RNA molecule) were set up according to experimental findings on the Mg^2+^ dependence of Gsw^loop^ properties [15,18,31]. Due to slow exchange times of first shell ligands of the Mg^2+^ ions [37], the Mg^2+^ ions were added to the RNA system with a first hydration shell of six water molecules. This was done to prevent an early “sticking” of the Mg^2+^ ions to the RNA during the equilibration phase. To prevent clashes between the hydration shell and the RNA during the placement step, dummy ions with equal charge but larger radius of 4 Å, which is close to the radius of a hexahydrated Mg^2+^ ion [87], were placed first at electrostatically favorable locations with *leap* of Amber 11; these dummy ions were then replaced with a hexahydrated Mg^2+^ ion [69]. For details about the influence of the initial placement and the choice of the Mg^2+^ parameters see [33]. The system was afterwards placed in an octahedral box of TIP3P water molecules [88]. The distance between the edge of the water box and the closest RNA atom was at least 11 Å. Na^+^ ions [89] were added to neutralize the system net charge, resulting in a final system size of ~50,000 atoms.

Each system was then prepared based on a protocol described earlier [90]. In detail, each system was minimized by 200 steps of steepest descent minimization, followed by 50 steps of conjugate gradient minimization. The particle mesh Ewald method [91] was used to treat long range electrostatic interactions, and the SHAKE algorithm [92] was used to constrain bond lengths involving bonds to hydrogen atoms. The time step for all MD simulations was 2 fs, with a direct-space nonbonded cutoff of 9 Å. We carried out canonical ensemble (NVT)-MD simulations for 50 ps, during which the system was heated from 100 to 300K. During this step, harmonic restraints with force constants of 5 kcal mol^-1^ Å^-2^ to all solute atoms were applied. In addition, harmonic restraints were applied to the Mg^2+^ ions and the water molecules in their first hydration shell. Subsequently, isothermal isobaric ensemble (NPT)-MD simulations were used for 50 ps to adjust the solvent density. Finally, the force constants of the harmonic restraints on positions of RNA, Mg^2+^, and first hydration shell waters were gradually reduced to 1 kcal mol^-1^ Å^-2^ during 250 ps of NVT-MD simulations. This was followed by 50 ps of NVT-MD simulations without applying positional restraints. From the following 550 ns of NVT-MD simulations at 300 K performed by the GPU version of *pmemd* [93], conformations were extracted every 20 ps.

For each of the six system setups (Gsw^apt^ and Gsw^loop^ with three Mg^2+^ concentrations each), three independent MD simulations of 550 ns length each were performed summing up to a total simulation time of ~10 μs.

### Trajectory analyses

The MD simulations were analyzed using *cpptraj* [94] of the AmberTools 13 program suite [95]. The initial 50 ns of each trajectory were discarded because the largest increase in overall RMSD was observed during this period (S3 Fig). The *radius of gyration* (ROG), a measure of the compactness of a structure, was calculated omitting the P1 region. This was done for conformations generated by MD simulations as well as for the crystal structures. Prior to the calculation of *root mean-square atomic fluctuations* (RMSF), global translational and rotational differences between the structures along the trajectory need to be removed by least-squares fitting. In order to reduce the influence of very mobile regions on the picture of internal motions [52,96–98], conformations were root mean-square fitted on those 80% of the nucleotides with the lowest fluctuations (S2 Table). These 54 nucleotides were chosen from an initial calculation of the mean RMSF over the three simulations of Gsw^apt^ in the absence of Mg^2+^ ions. These nucleotides were also used in the case of Gsw^loop^ then. *Root mean-square deviations* (RMSD) of atomic positions were calculated as a measure for structural similarity with respect to the initial structures. The RMSD was calculated after aligning the conformations onto the initial structure, using those 80% of the nucleotides with the lowest RMSF. The RMSD was then calculated for all atoms of the RNA as well as for all atoms of substructural parts of the aptamer (S5 Table). In order to investigate the change within a substructural part of the aptamer, RMSD values in S3 Table were calculated for the substructural parts after root mean-square fitting on the respective part in the initial conformation. Mean values were calculated over all three MD trajectories. Preferred *Mg*^*2+*^ *ion occupancy sites* were determined by the *cpptraj* program using the *grid* command using a grid spacing of 0.4 Å. The *occupancy for hydrogen bonds* between bases in the L2/L3 base quadruples were determined using default geometrical parameters (distance: 3.5 Å; angle: 120°). Block averaging over blocks of 10 ns length was used to determine the statistical uncertainty within one trajectory. The values reported in Table 2 were averaged over all hydrogen bonds between a pair of bases. The hexahydration of Mg^2+^ ions was determined by “following” the hydration shell around the ions over the simulation. Here, “following” means that we calculated the number of water molecules around each Mg^2+^ ion within a distance of 3.5 Å. Changes in the number of water molecules in the first hydration shell were subsequently confirmed by visual inspection. Furthermore, the direct chelation of Mg^2+^ ions to the RNA was determined by calculating the distance of each Mg^2+^ ion to the RNA. We considered a distance below 3.5 Å between a Mg^2+^ ion and an RNA atom indicative that the hydration shell of this Mg^2+^ ion is incomplete. The direct chelation was then confirmed by visual inspection of the trajectory.

We assessed the convergence of our simulations in two ways: First, we performed a principal component analysis for the phosphorus atoms of the aptamer over all trajectories. Then, we projected each trajectory onto the combinations of principal components (PC) PC1/PC2, PC1/PC3, and PC2/PC3. As shown in S10–S12 Figs exemplarily for Gsw^apt^ in the presence of 20 Mg^2+^ ions and Gsw^loop^ in the absence of Mg^2+^, all projections markedly overlap when comparing MD simulations of the same system, and when comparing the first and the second half of the trajectories. For Gsw^loop^ in the absence of Mg^2+^ ions, the projections onto PC1 show a larger spread between the MD simulations; however, the projections onto PC2 and PC3 overlap well. These results demonstrate that the three independent MD simulations per system sample similar regions in PC space, and the sampling agrees between the first and second halves of the trajectories. Additionally, we calculated the RMSD average correlation (RAC) [94,99] as implemented in *cpptraj* [94]. The RAC is a measure for the time scales on which structural changes happen in MD simulations. The RAC curves for all our simulations are shown in S13 Fig. From the bumps in the curves, we can estimate that structural changes happen on time scales of ~50–100 ns for Gsw^loop^ in the absence of Mg^2+^ ions (S13B Fig), whereas for the other systems, such bumps are found at shorter time intervals. For time intervals > 300 ns, the curves appear smooth, which suggests that there are no large structural changes happening on these time scales. Note that we calculated the RAC with respect to the first structure omitting the first 50 ns of each MD trajectory. As an alternative, the RAC can be calculated with respect to the average structure of an MD trajectory, which would result in smaller values [99]. Overall, these analyses are indicative to what extent the conformational ensembles of sampled aptamer structures are converged.

### Rigidity analyses

In order to investigate the influence of the presence of a ligand in the binding site on the rigidity characteristics of the aptamer domain, we applied the FIRST software (version 6.2) [48]. FIRST converts structural information of an input structure into a constraint network representation, where vertices represent the atoms and edges represent interatomic interactions, e.g. covalent bonds, hydrogen bonds, or hydrophobic interactions. FIRST then uses the pebble game algorithm [49,50] to decompose the network into rigid regions and flexible links.

FIRST has not only been successfully applied to protein structures, but also proved successful in investigating and predicting flexibility and rigidity characteristics of RNA structures [56–59]. Here, we use an RNA-specific set of parameters developed by Fulle *et al*. [59] for modeling constraints due to non-covalent interactions, using -0.6 kcal mol^-1^ as an energy cutoff *E*_cut_ for hydrogen bonds (Fig 3C, 3D, 3E and 3F; S9 Fig). A value of *E*_*cut*_ = −1.0 *kcal*/*mol* yielded very similar results (S14 Fig), as found earlier [59].

From the rigid cluster decomposition, we calculate the probability *p*_*lrc*_(*i*) that atom *i* belongs to the largest rigid cluster according to $p_{lrc}\left(i\right)=\;\frac{n_{1}\left(i\right)}{N}$, where *n*_1_(*i*) is the number of occurrences of atom *i* as part of the largest rigid cluster, determined over all *N* snapshots extracted from the trajectory [52]. *p*_*lrc*_(*i*) was calculated in the absence (*apo*) and presence (*lig*) of a ligand, as was the difference

$$\Delta p_{lrc}\left(i\right)=\;p_{lrc}{\left(i\right)}_{lig}-\;p_{lrc}{\left(i\right)}_{apo}$$ (1)

The average of Δ*p*_*lrc*_(*i*) over the three independent MD simulations for each of the six system setups is shown in S9 Fig, and projected onto the aptamer domain in Fig 3C, 3D, 3E and 3F. For the visualization, the value calculated for the N1 atom of each nucleotide's base is projected onto the whole nucleotide.

Averaging over an ensemble of snapshots from an MD simulation increases the robustness of the results compared to using a single input structure only [52,60,61]. Snapshots were extracted from the MD trajectories, considering the RNA and, if present, Mg^2+^ ions and their first hydration shell water molecules as proposed by Fulle *et al*. [59]. In detail, interactions between Mg^2+^ ions and their first hydration shell water molecules, or Mg^2+^ ions and the RNA, were modeled as covalent bonds, whereas interactions between water and RNA were modeled as hydrogen bonds. These snapshots were subsequently converted using the *ambpdb* program from the AmberTools suite of programs to FIRSTdataset files. In order to model the presence of the ligand guanine for the rigidity analyses by FIRST, we added constraints to the FIRSTdataset file connecting nucleotides U47, C74, and U51 in a pair-wise manner; these nucleotides interact with the ligand in the crystal structure (Fig 3A and 3B). To validate that the added constraints represent the bound ligand in the binding site, we additionally performed the rigidity analysis on the minimized X-ray structure of Gsw^loop^ (i) without a ligand in the binding site, (ii) with guanine at the position of the original ligand, and (iii) with the added constraints representing the ligand, and investigated the rigid cluster decomposition of the binding nucleotides (U47, U51, and C74) (S15 Fig). Without guanine or the ligand-representing constraints, the binding nucleotides do not belong to one rigid cluster (S15A Fig). The presence of guanine at the ligand binding site results in a rigid cluster that comprises all three binding nucleobases (S15B Fig). Similarly, when adding the ligand-representing constraints to the network, all three binding nucleobases are part of one rigid cluster (S15C Fig). The difference in the spread of the rigid cluster visible between S15B and S15C Fig relates to using a “weak” coupling scheme for the ligand-representing constraints, as discussed above.

In order to investigate the interplay between the presence of tertiary interactions in the loop region and the presence of a ligand in the binding site, we evaluate cooperative effects on the P1 region. To do so, we computed *p*_*lrc*_(*i*) values for the aptamer domain without ligand constraints ($p_{lrc}{\left(i\right)}_{apo}^{Gsw^{apt}}$ and $p_{lrc}{\left(i\right)}_{apo}^{Gsw^{loop}}$ for Gsw^apt^ and Gsw^loop^, respectively) and with ligand constraints ($p_{lrc}{\left(i\right)}_{lig}^{Gsw^{apt}}$ and $p_{lrc}{\left(i\right)}_{lig}^{Gsw^{loop}}$ for Gsw^apt^ and Gsw^loop^, respectively). $p_{lrc}{\left(i\right)}_{apo}^{Gsw^{loop}}$ was chosen as the reference because it lacks interactions at both the loop region and the binding site. From this, we calculated the cooperative effect on the stabilization of the P1 region due to interactions at the two sites according to

$$Coop\left(i\right)=ln\left(\frac{p_{lrc}{\left(i\right)}_{lig}^{Gsw^{apt}}}{p_{lrc}{\left(i\right)}_{apo}^{Gsw^{loop}}}\right)-\;\left[ln\left(\frac{p_{lrc}{\left(i\right)}_{apo}^{Gsw^{apt}}}{p_{lrc}{\left(i\right)}_{apo}^{Gsw^{loop}}}\right)+ln\left(\frac{p_{lrc}{\left(i\right)}_{lig}^{Gsw^{loop}}}{p_{lrc}{\left(i\right)}_{apo}^{Gsw^{loop}}}\right)\right]$$ (2)

where the term

$$ln\left(\frac{p_{lrc}{\left(i\right)}_{lig}^{Gsw^{apt}}}{p_{lrc}{\left(i\right)}_{apo}^{Gsw^{loop}}}\right)$$ (3)

describes the influence of the tertiary interactions together with the ligand,

$$ln\left(\frac{p_{lrc}{\left(i\right)}_{apo}^{Gsw^{apt}}}{p_{lrc}{\left(i\right)}_{apo}^{Gsw^{loop}}}\right)$$ (4)

describes the influence of the tertiary interactions alone, and

$$ln\left(\frac{p_{lrc}{\left(i\right)}_{lig}^{Gsw^{loop}}}{p_{lrc}{\left(i\right)}_{apo}^{Gsw^{loop}}}\right)$$ (5)

describes the influence of the ligand alone.

If *Coop*(*i*) > 0, interactions at both sites together have a larger effect on nucleotide *i* than the sum of the effects of the separate interactions. We are mainly interested in the effect on the terminal nucleotides in the P1 region, and thus in *Coop*(*i*) for *i* ∈ {15, 16, 17, 18, 19, 77, 78, 79, 80, 81}.

## Supporting information

S1 Fig Positions of three Mg^2+^ ions over a simulation time of 550 ns. The ions (colored in blue, green, and orange) explore the space around the Gsw aptamer (black) in a MD simulation of Gsw^apt^ with 20 Mg^2+^ ions.
(TIFF)

S2 Fig Number of hexahydrated Mg^2+^ over the simulation time. Results are shown for three simulations of Gsw^apt^ (three blue, solid lines) and Gsw^loop^ (three red, dashed lines). A: Simulations with 12 Mg^2+^ ions; B: Simulations with 20 Mg^2+^ ions.
(TIFF)

S3 Fig RMSD of all nucleotides of the aptamer with respect to the first conformation after fitting of the 80% least fluctuating nucleotides (S2 Table). The RMSD values for the three independent simulations are shown in different grey colors for Gsw^apt^ in the absence of Mg^2+^ (A) and the presence of 20 Mg^2+^ (B).
(TIFF)

S4 Fig Average conformation calculated over the last 50 ns of the MD simulations. The average conformations for Gsw^apt^ in the absence of Mg^2+^ (A) and in the presence of 20 Mg^2+^ ions (B) and for Gsw^loop^ in the absence of Mg^2+^ (C) and in the presence of 20 Mg^2+^ ions (D) (colored in different grey tones for the three independent simulations) is overlaid with the crystal structure of Gsw^apt^ (PDB ID 4FE5 [17]) for (A) and (B) and of Gsw^loop^ (PDB ID 3RKF [18]) for (C) and (D) (colored in blue).
(TIFF)

S5 Fig Distribution of χ dihedral for different force fields. Normalized histogram for simulations of simulations of Gsw^apt^ (A) and Gsw^loop^ (B) in the presence of 20 Mg^2+^ ions using the ff99 force field (blue) and the ff10 force field (red) for comparison. Different line types correspond to three independent simulations. Grey boxes correspond to χ dihedrals in the crystal structures with PDB ID 4FE5 [17] and 3RKF [18]. The simulations using the ff10 force field were extended from our previous study [33] to a simulation length 550 ns each.
(TIFF)

S6 Fig Distribution of χ dihedrals in the P1 region of the aptamer for different force fields. Normalized histogram for simulations of Gsw^apt^ (A) and Gsw^loop^ (B) in the presence of 20 Mg^2+^ ions using the ff99 force field (blue) and the ff10 force field (red) for comparison. Different line types correspond to three independent simulations. Grey boxes correspond to **χ** dihedrals in the crystal structures with PDB ID 4FE5 (17) and 3RKF (18). The simulations using the ff10 force field were extended from our previous study [33] to a simulation length 550 ns each.
(TIFF)

S7 Fig Lower base quadruple formed by the L2 and L3 loops. A: Gsw^apt^; B: Gsw^loop^. Hydrogen bonds are shown as black dashed lines. Bases are colored as in Fig 1A according to which loop they belong to.
(TIFF)

S8 Fig Differences in RMSF in the presence of 20 Mg^2+^ ions for Gsw^loop^—Gsw^apt^ projected onto the aptamer structure. Larger differences are colored red, smaller differences blue. Nucleotides responsible for ligand binding are shown as sticks.
(TIFF)

S9 Fig Average difference (±SEM) in the probability (Δ*p*_*lrc*_(*i*), Eq 1) of each nucleotide to be in the largest rigid cluster between the ligand being present or absent in the aptamer domain, using a value of *E*_*HB*_ = −0.6 kcal/mol for the rigidity analyses. The differences are shown in the absence of Mg^2+^ ions (red) or in the presence of 12 (green) and 20 (blue) Mg^2+^ ions for Gsw^apt^ (A) and Gsw^loop^ (B). The colored boxes on the top depict substructures of the riboswitch.
(TIFF)

S10 Fig Projection of snapshots onto PC1 and PC2. The principal component analysis was performed over all MD trajectories. The trajectories for Gsw^apt^ with 20 Mg^2+^ ions (A, C) and Gsw^loop^ in the absence of Mg^2+^ ions (B, D) are projected onto the first (PC1) and second (PC2) eigenvector. The projections for the first (A, B) and second half (C, D) of the trajectories are shown separately. Histograms are shown on top and on the right side of the projections. Different blue and red colors correspond to the three independent MD simulations of each system.
(TIFF)

S11 Fig Projection of snapshots onto PC1 and PC3. The principal component analysis was performed over all MD trajectories. The trajectories for Gsw^apt^ with 20 Mg^2+^ ions (A, C) and Gsw^loop^ in the absence of Mg^2+^ ions (B, D) are projected onto the first (PC1) and third (PC3) eigenvector. The projections for the first (A, B) and second half (C, D) of the trajectories are shown separately. Histograms are shown on top and on the right side of the projections. Different blue and red colors correspond to the three independent MD simulations of each system.
(TIFF)

S12 Fig Projection of snapshots onto PC2 and PC3. The principal component analysis was performed over all MD trajectories. The trajectories for Gsw^apt^ with 20 Mg^2+^ ions (A, C) and Gsw^loop^ in the absence of Mg^2+^ ions (B, D) are projected onto the second (PC2) and third (PC3) eigenvector. The projections for the first (A, B) and second half (C, D) of the trajectories are shown separately. Histograms are shown on top and on the right side of the projections. Different blue and red colors correspond to the three independent MD simulations of each system.
(TIFF)

S13 Fig RMSD average correlation (RAC). The RAC for trajectories of Gsw^apt^ in the absence (A), with 12 Mg^2+^ (C), and 20 Mg^2+^ ions (E), and of Gsw^loop^ in the absence (B), with 12 Mg^2+^ (D), and 20 Mg^2+^ ions (F) is shown for time intervals up to 500 ns. The RAC was calculated with respect to the first conformation, omitting the first 50 ns of each trajectory.
(TIFF)

S14 Fig Differences in the probability of each nucleotide to be in the largest rigid cluster between the ligand being present or absent in the aptamer domain, using a value of *E*_*HB*_ = −1.0 kcal/mol for the rigidity analyses. A, B: Average difference (±SEM). The differences are shown in the absence of Mg^2+^ ions (red) or in the presence of 12 (green) and 20 (blue) Mg^2+^ ions for Gsw^apt^ (A) and Gsw^loop^ (B). The colored boxes on the top depict substructures of the riboswitch. C, D, E, F: Average difference in the probability (Δ*p*_*lrc*_(*i*), Eq 1) of each nucleotide to be in the largest rigid cluster between the ligand being present or absent in the aptamer domain, projected onto the aptamer domain of Gsw^apt^ (C, E) and Gsw^loop^ (D, F) in the absence of Mg^2+^ ions (C, D) and in the presence of 20 Mg^2+^ ions (E, F).
(TIFF)

S15 Fig Rigid cluster decomposition of the ligand binding site (nucleotides U47, U51, C74). The blue sticks represent one rigid cluster obtained from the rigid cluster decomposition using the FIRST software. For this analysis, the X-ray structure of Gsw^loop^ was subjected to an energy minimization before the rigidity analysis. Here, only the binding nucleotides are shown. A: The binding site of Gsw^loop^ is empty. B: Guanine is present in the binding pocket. C: Constraints representing the bound ligand (c.f. Methods section "Rigidity analyses") were placed between the binding nucleotides.
(TIFF)

S1 Table Occupation of experimentally determined Mg^2+^ binding sites. (PDF)

S2 Table Core nucleotides taken into account for analysis. (PDF)

S3 Table Root mean square deviations of Gsw^apt^ and Gsw^loop^ for substructural parts of the aptamer. (PDF)

S4 Table Root mean square fluctuations for Gsw^apt^ and Gsw^loop^ for the 80% least fluctuating nucleotides ("core nucleotides"). (PDF)

S5 Table Assignment of nucleotides to substructural parts of the Gsw aptamer domain. (PDF)
