# Supplementary figures and images for: Ligand-mediated and tertiary interactions cooperatively stabilize the P1 region in the guanine-sensing riboswitch
Source: PLoS One. 2017 Jun 22;12(6):e0179271. doi: 10.1371/journal.pone.0179271 (PMC5480868; doi:10.1371/journal.pone.0179271)

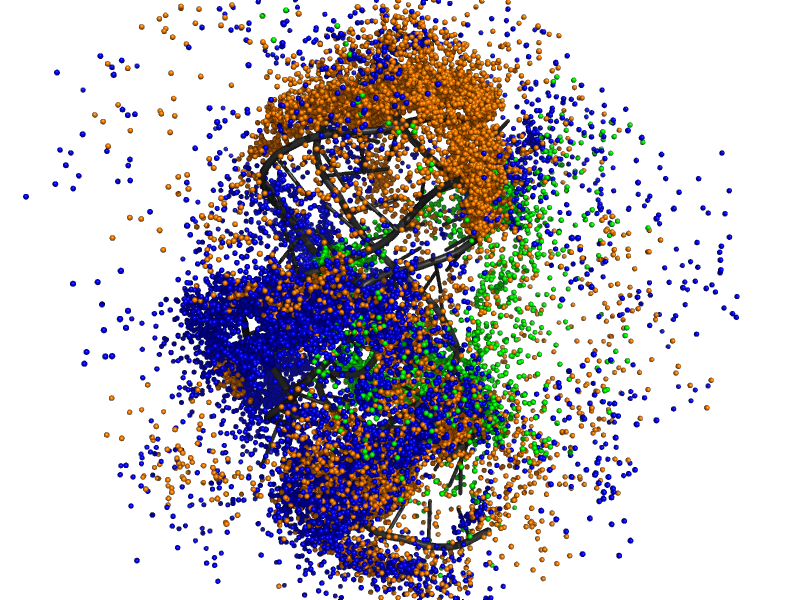

Supplement: S1 Fig — The ions (colored in blue, green, and orange) explore the space around the Gsw aptamer (black) in a MD simulation of Gswapt with 20 Mg2+ ions. (TIFF) [file pone.0179271.s001.tiff]

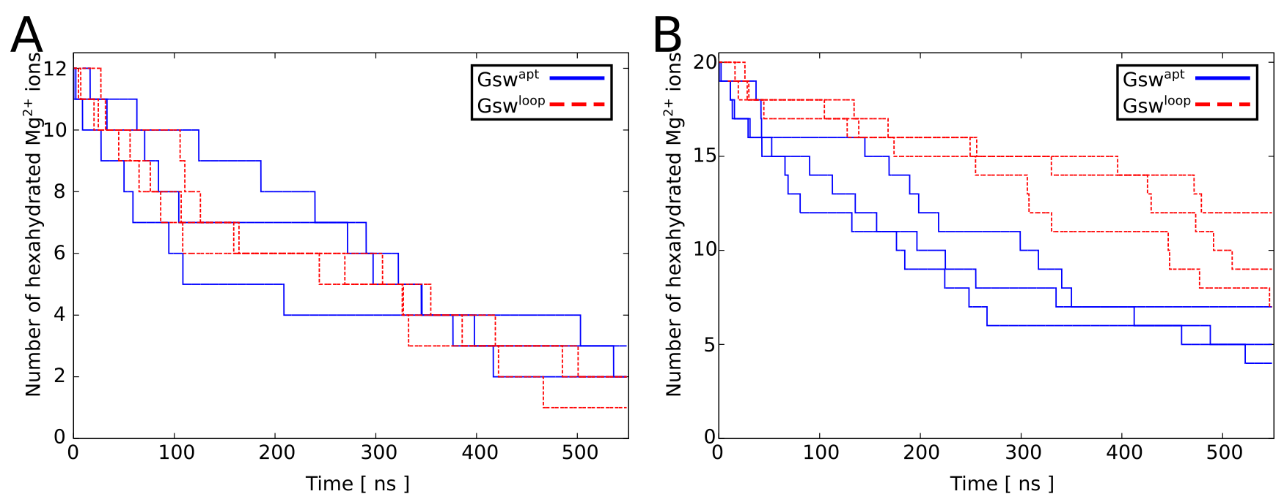

Supplement: S2 Fig — Results are shown for three simulations of Gswapt (three blue, solid lines) and Gswloop (three red, dashed lines). A: Simulations with 12 Mg2+ ions; B: Simulations with 20 Mg2+ ions. (TIFF) [file pone.0179271.s002.tiff]

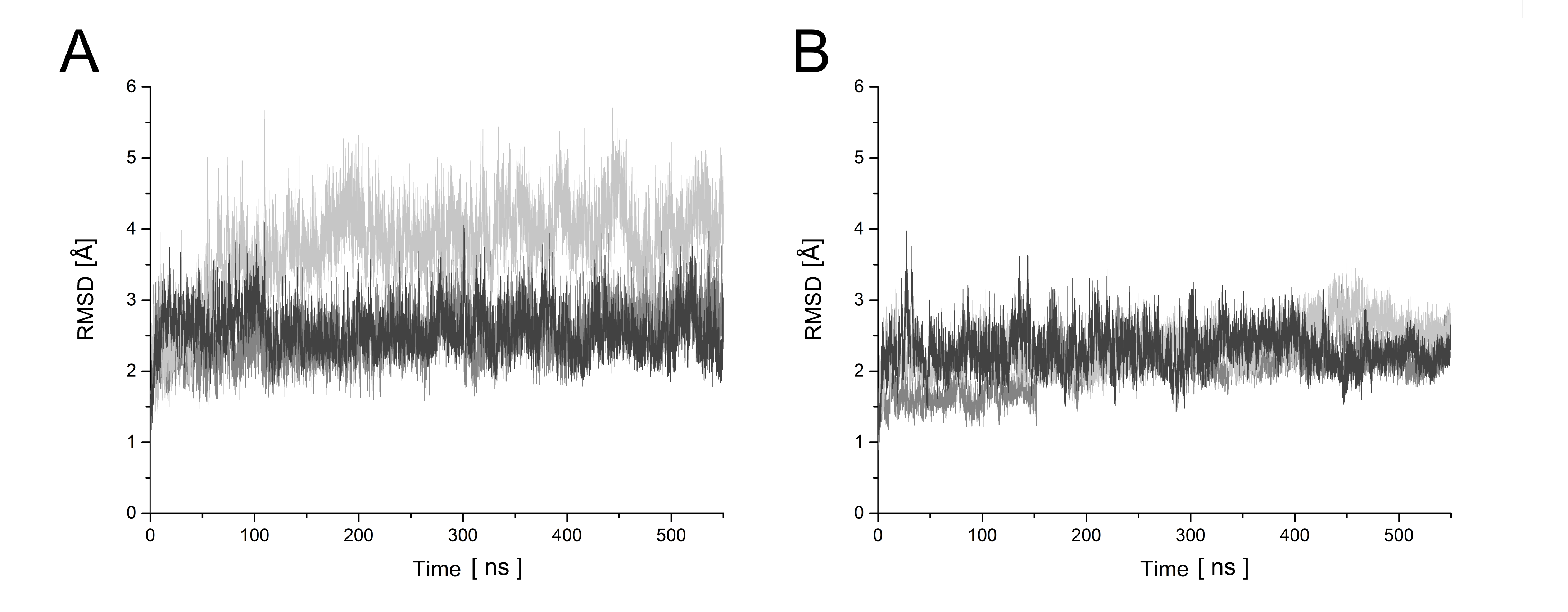

Supplement: S3 Fig — The RMSD values for the three independent simulations are shown in different grey colors for Gswapt in the absence of Mg2+ (A) and the presence of 20 Mg2+ (B). (TIFF) [file pone.0179271.s003.tiff]

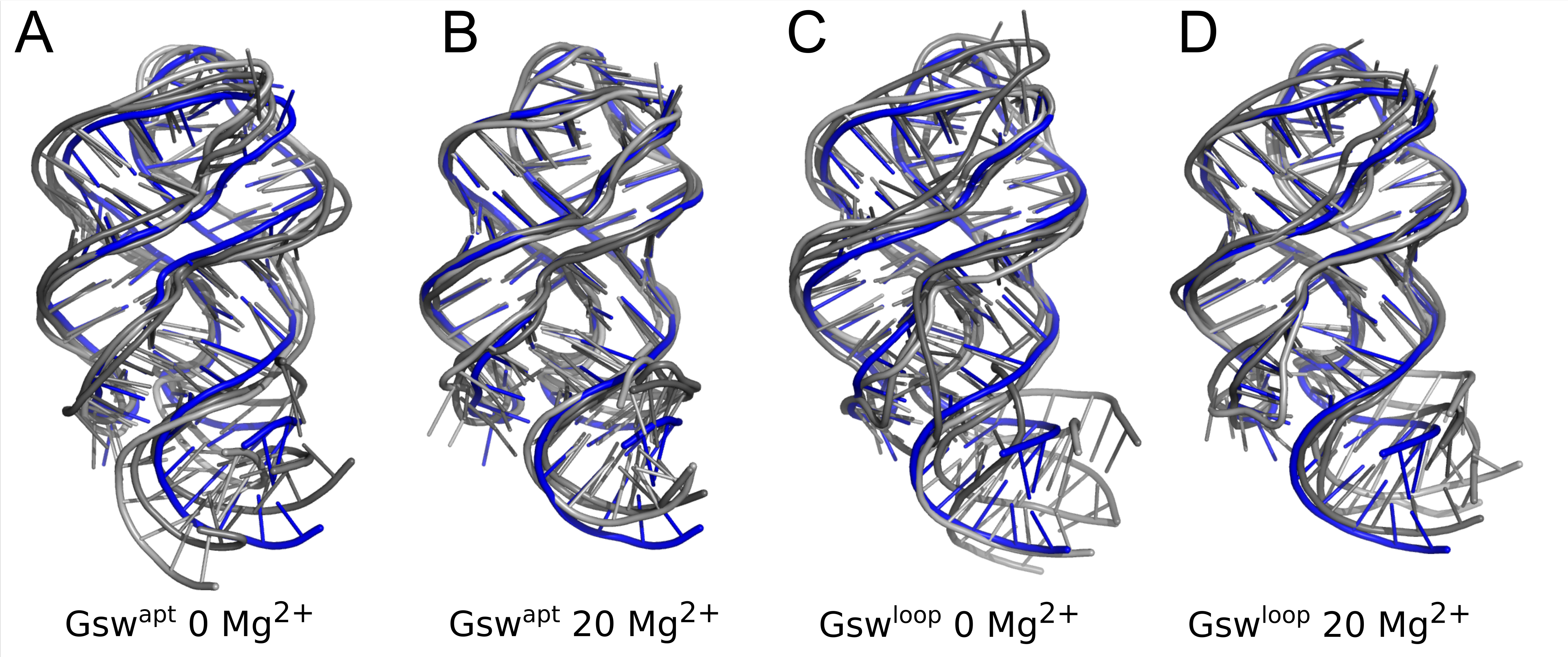

Supplement: S4 Fig — The average conformations for Gswapt in the absence of Mg2+ (A) and in the presence of 20 Mg2+ ions (B) and for Gswloop in the absence of Mg2+ (C) and in the presence of 20 Mg2+ ions (D) (colored in different grey tones for the three independent simulations) is overlaid with the crystal structure of Gswapt (PDB ID 4FE5 [17]) for (A) and (B) and of Gswloop (PDB ID 3RKF [18]) for (C) and (D) (colored in blue). (TIFF) [file pone.0179271.s004.tiff]

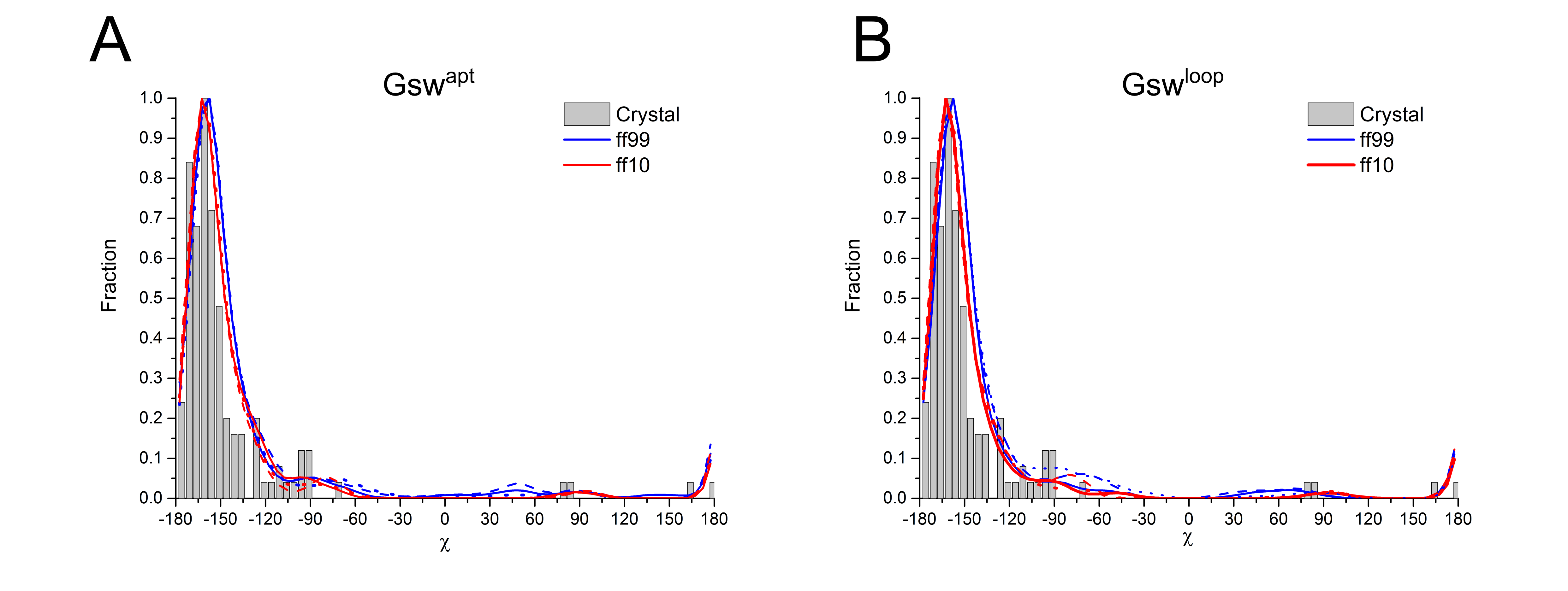

Supplement: S5 Fig — Normalized histogram for simulations of simulations of Gswapt (A) and Gswloop (B) in the presence of 20 Mg2+ ions using the ff99 force field (blue) and the ff10 force field (red) for comparison. Different line types correspond to three independent simulations. Grey boxes correspond to χ dihedrals in the crystal structures with PDB ID 4FE5 [17] and 3RKF [18]. The simulations using the ff10 force field were extended from our previous study [33] to a simulation length 550 ns each. (TIFF) [file pone.0179271.s005.tiff]

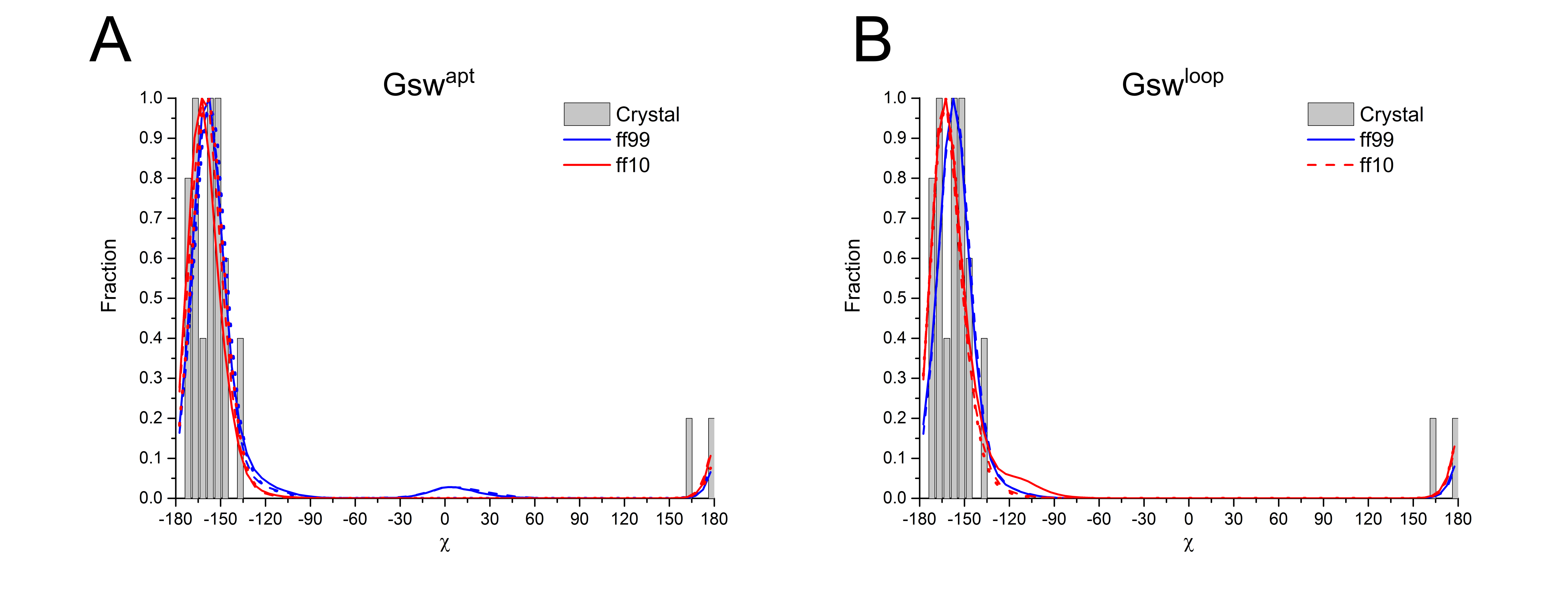

Supplement: S6 Fig — Normalized histogram for simulations of Gswapt (A) and Gswloop (B) in the presence of 20 Mg2+ ions using the ff99 force field (blue) and the ff10 force field (red) for comparison. Different line types correspond to three independent simulations. Grey boxes correspond to χ dihedrals in the crystal structures with PDB ID 4FE5 (17) and 3RKF (18). The simulations using the ff10 force field were extended from our previous study [33] to a simulation length 550 ns each. (TIFF) [file pone.0179271.s006.tiff]

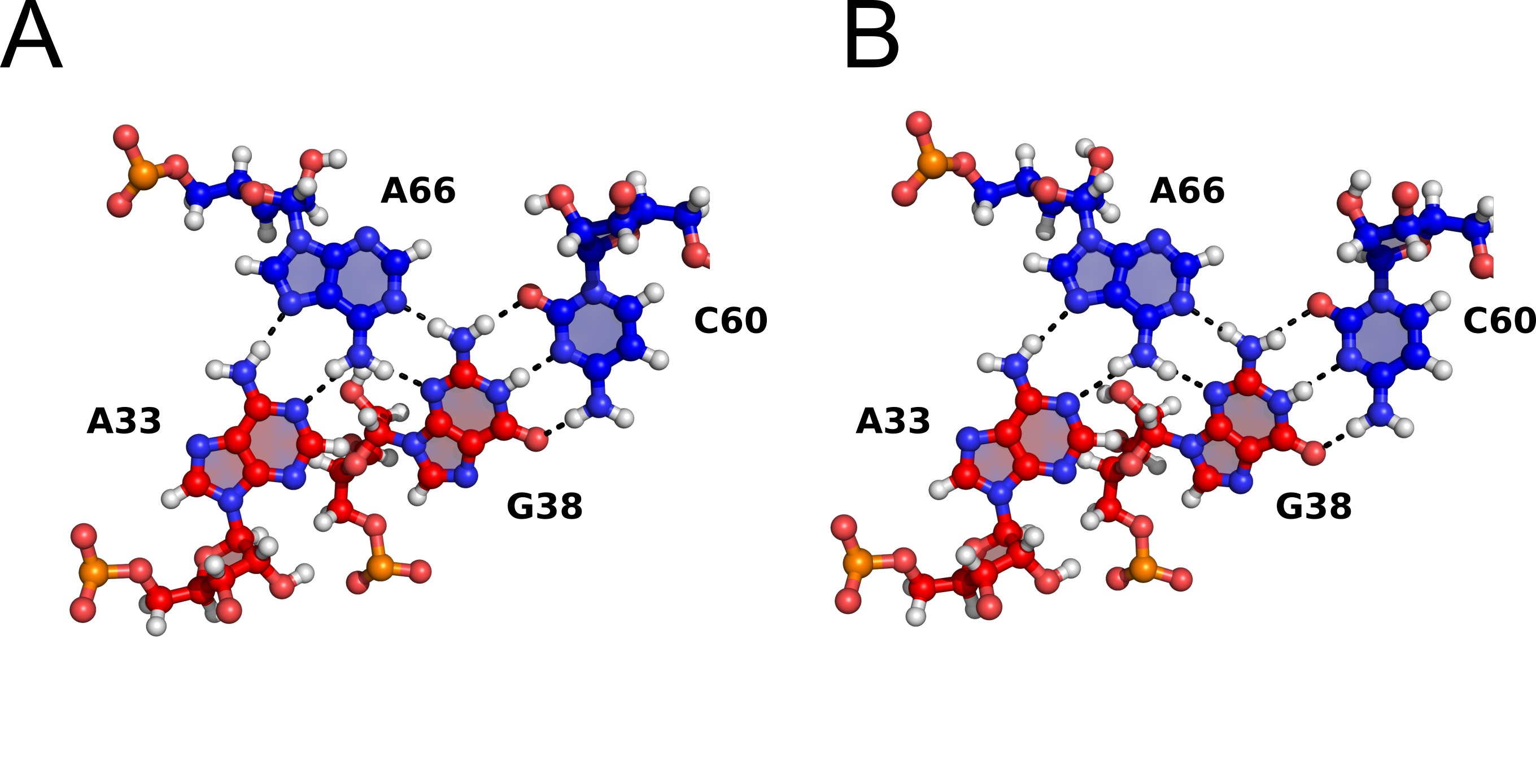

Supplement: S7 Fig — A: Gswapt; B: Gswloop. Hydrogen bonds are shown as black dashed lines. Bases are colored as in Fig 1A according to which loop they belong to. (TIFF) [file pone.0179271.s007.tiff]

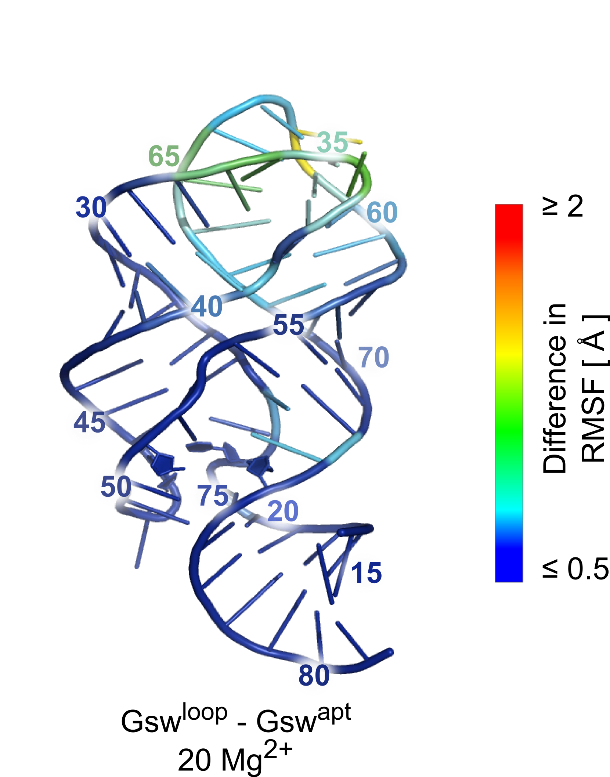

Supplement: S8 Fig — Larger differences are colored red, smaller differences blue. Nucleotides responsible for ligand binding are shown as sticks. (TIFF) [file pone.0179271.s008.tiff]

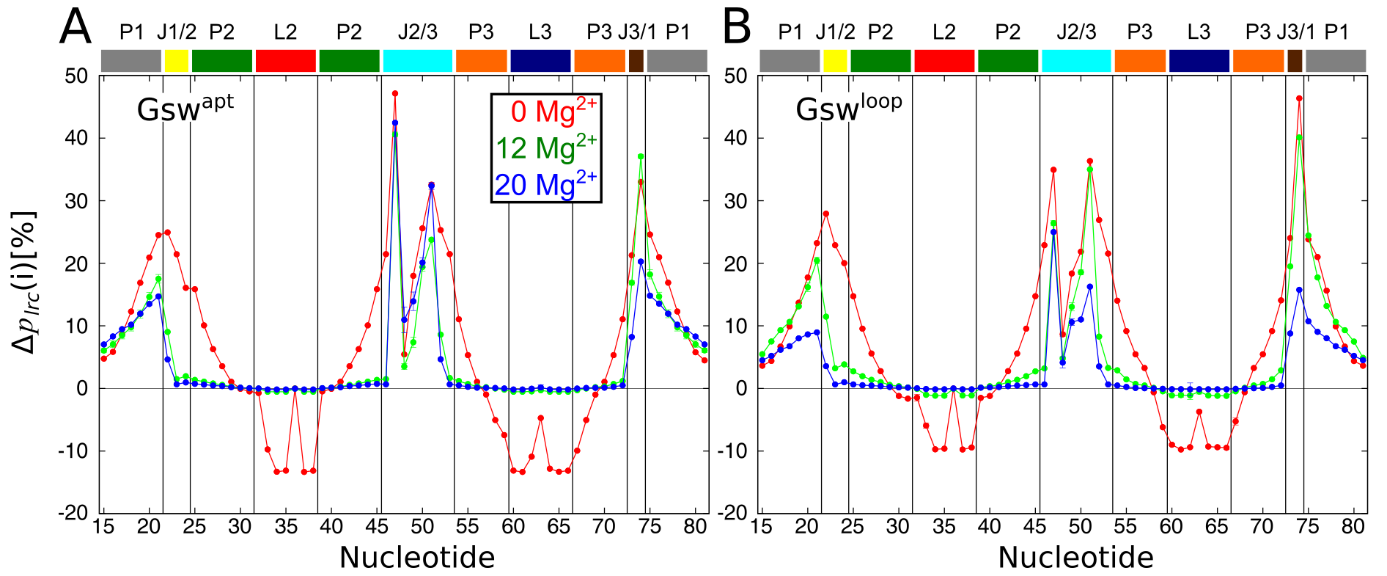

Supplement: S9 Fig — The differences are shown in the absence of Mg2+ ions (red) or in the presence of 12 (green) and 20 (blue) Mg2+ ions for Gswapt (A) and Gswloop (B). The colored boxes on the top depict substructures of the riboswitch. (TIFF) [file pone.0179271.s009.tiff]

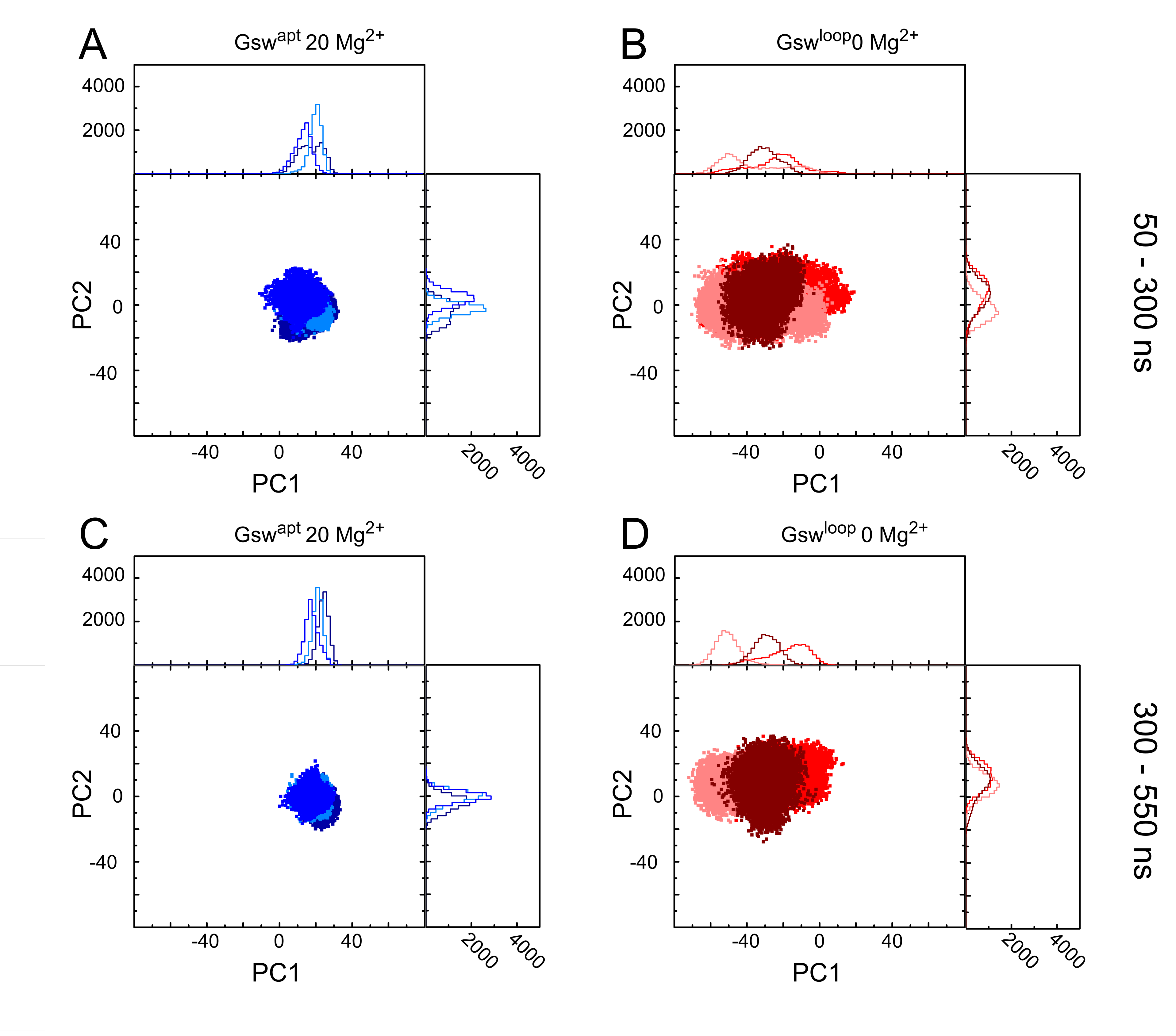

Supplement: S10 Fig — The principal component analysis was performed over all MD trajectories. The trajectories for Gswapt with 20 Mg2+ ions (A, C) and Gswloop in the absence of Mg2+ ions (B, D) are projected onto the first (PC1) and second (PC2) eigenvector. The projections for the first (A, B) and second half (C, D) of the trajectories are shown separately. Histograms are shown on top and on the right side of the projections. Different blue and red colors correspond to the three independent MD simulations of each system. (TIFF) [file pone.0179271.s010.tiff]

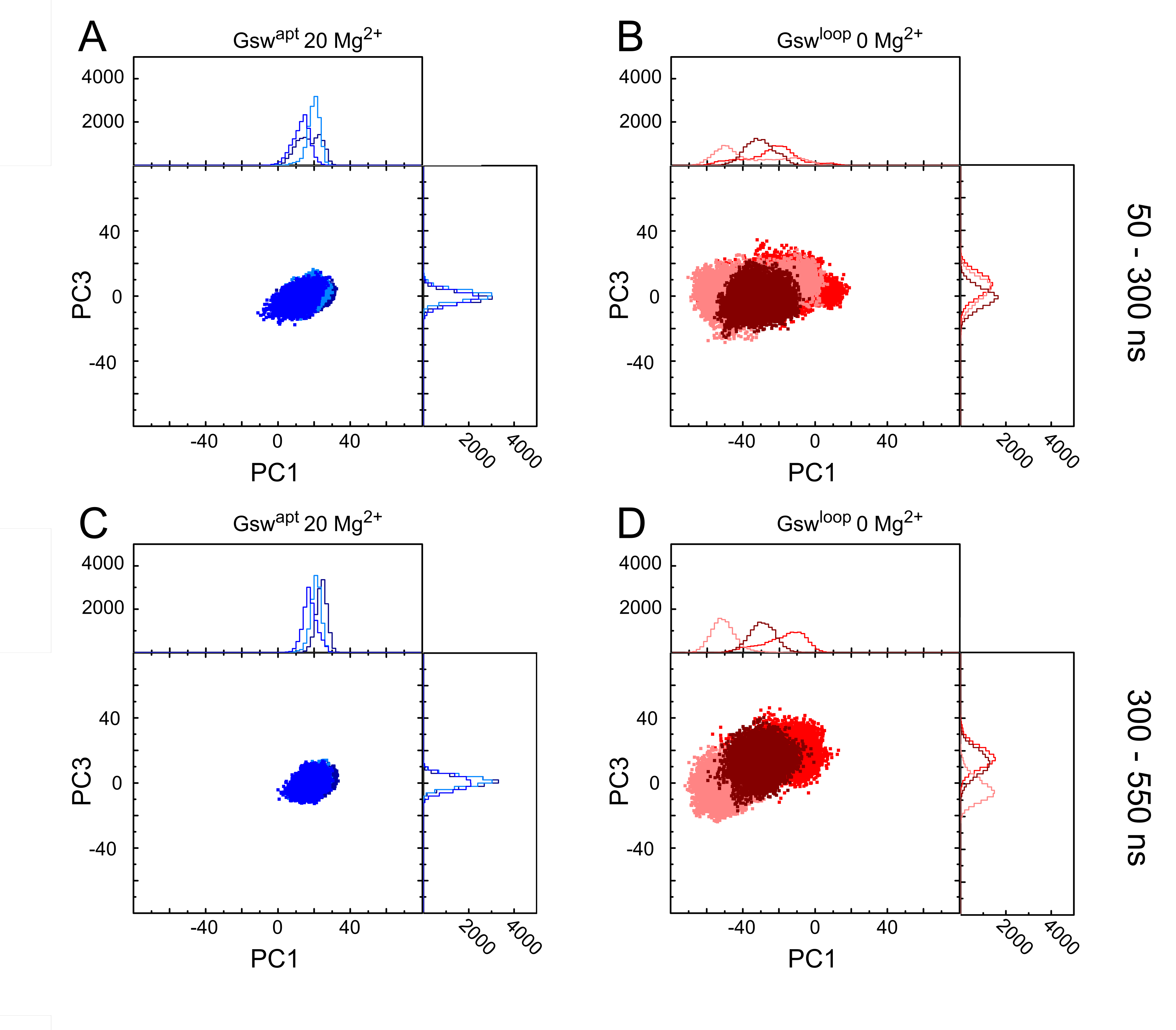

Supplement: S11 Fig — The principal component analysis was performed over all MD trajectories. The trajectories for Gswapt with 20 Mg2+ ions (A, C) and Gswloop in the absence of Mg2+ ions (B, D) are projected onto the first (PC1) and third (PC3) eigenvector. The projections for the first (A, B) and second half (C, D) of the trajectories are shown separately. Histograms are shown on top and on the right side of the projections. Different blue and red colors correspond to the three independent MD simulations of each system. (TIFF) [file pone.0179271.s011.tiff]

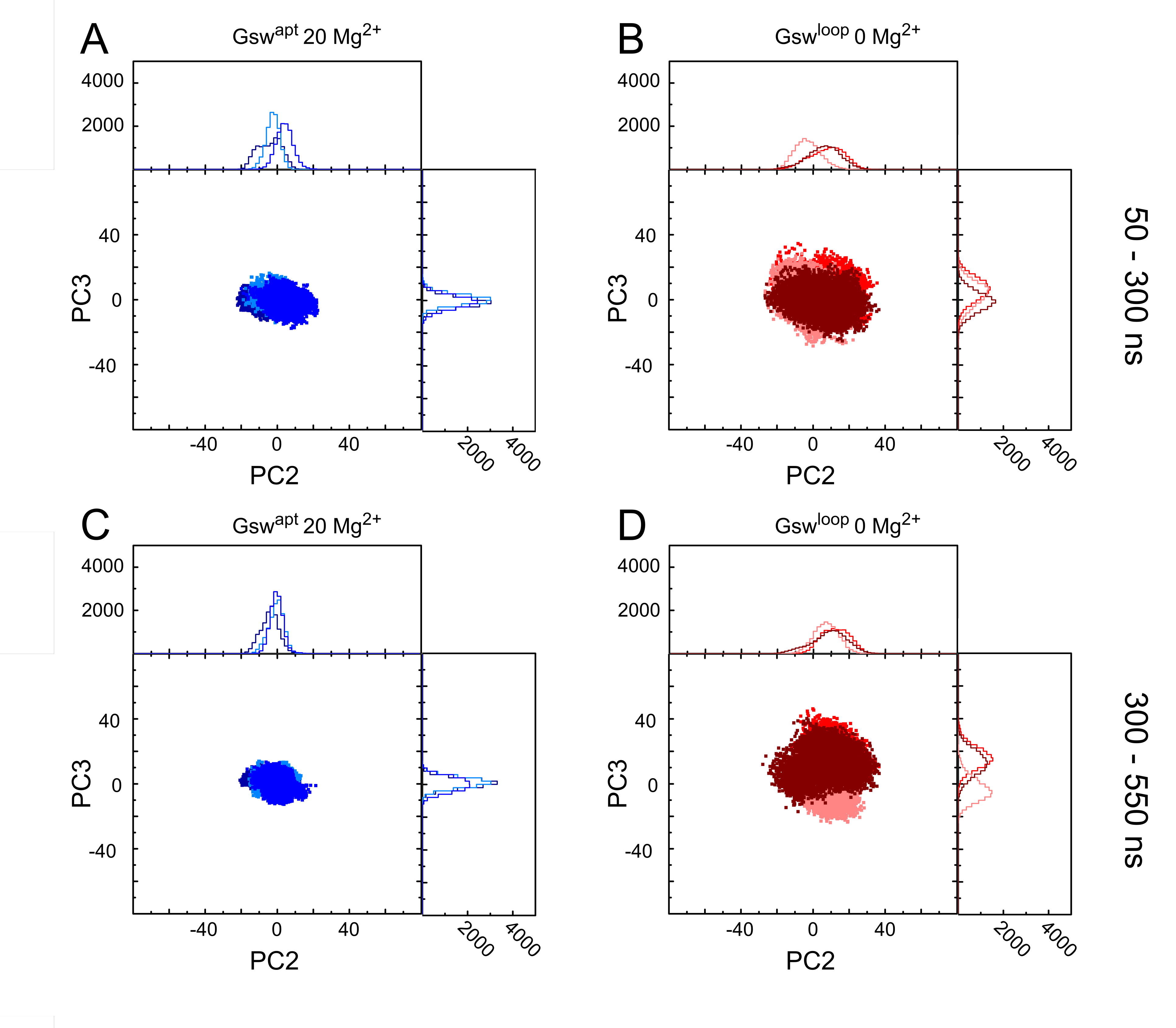

Supplement: S12 Fig — The principal component analysis was performed over all MD trajectories. The trajectories for Gswapt with 20 Mg2+ ions (A, C) and Gswloop in the absence of Mg2+ ions (B, D) are projected onto the second (PC2) and third (PC3) eigenvector. The projections for the first (A, B) and second half (C, D) of the trajectories are shown separately. Histograms are shown on top and on the right side of the projections. Different blue and red colors correspond to the three independent MD simulations of each system. (TIFF) [file pone.0179271.s012.tiff]

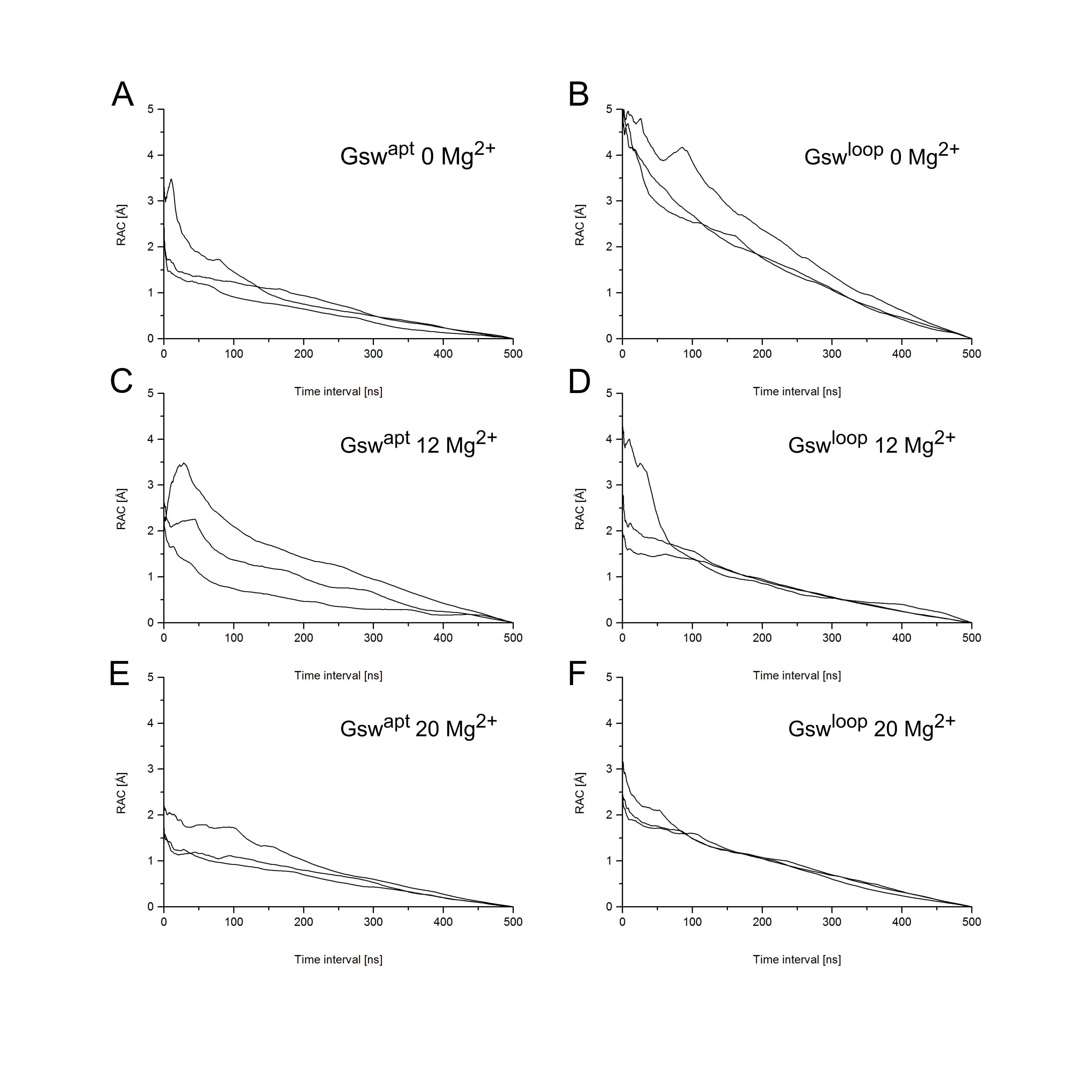

Supplement: S13 Fig — The RAC for trajectories of Gswapt in the absence (A), with 12 Mg2+ (C), and 20 Mg2+ ions (E), and of Gswloop in the absence (B), with 12 Mg2+ (D), and 20 Mg2+ ions (F) is shown for time intervals up to 500 ns. The RAC was calculated with respect to the first conformation, omitting the first 50 ns of each trajectory. (TIFF) [file pone.0179271.s013.tiff]

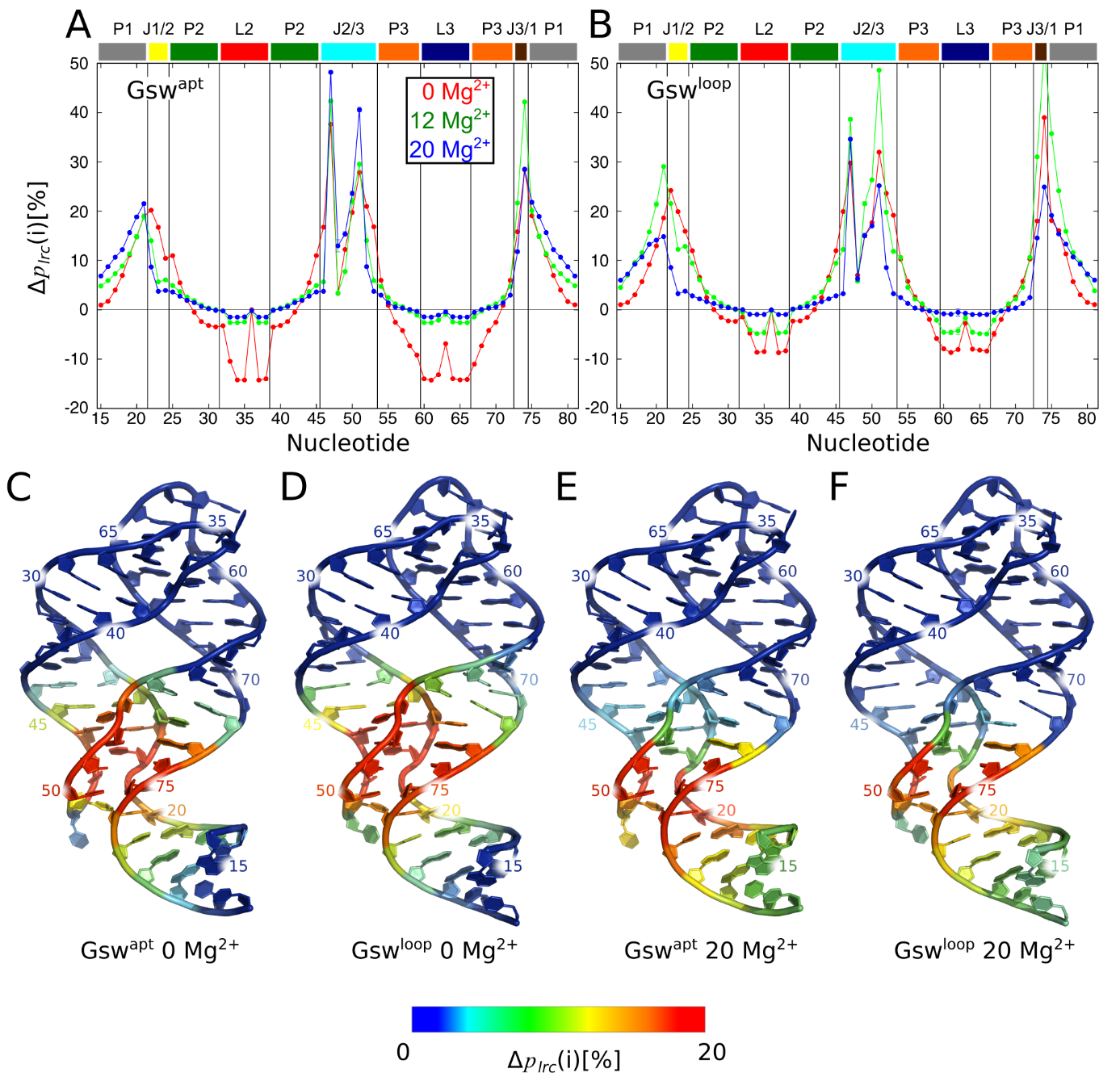

Supplement: S14 Fig — A, B: Average difference (±SEM). The differences are shown in the absence of Mg2+ ions (red) or in the presence of 12 (green) and 20 (blue) Mg2+ ions for Gswapt (A) and Gswloop (B). The colored boxes on the top depict substructures of the riboswitch. C, D, E, F: Average difference in the probability (Δplrc(i), Eq 1) of each nucleotide to be in the largest rigid cluster between the ligand being present or absent in the aptamer domain, projected onto the aptamer domain of Gswapt (C, E) and Gswloop (D, F) in the absence of Mg2+ ions (C, D) and in the presence of 20 Mg2+ ions (E, F). (TIFF) [file pone.0179271.s014.tiff]

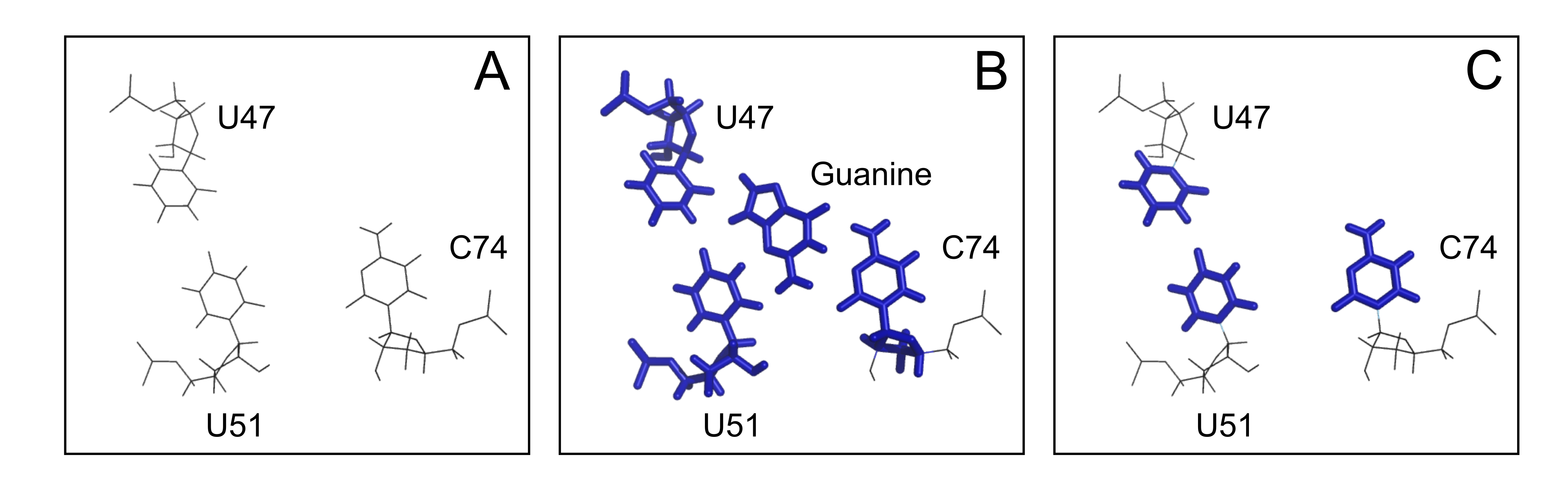

Supplement: S15 Fig — The blue sticks represent one rigid cluster obtained from the rigid cluster decomposition using the FIRST software. For this analysis, the X-ray structure of Gswloop was subjected to an energy minimization before the rigidity analysis. Here, only the binding nucleotides are shown. A: The binding site of Gswloop is empty. B: Guanine is present in the binding pocket. C: Constraints representing the bound ligand (c.f. Methods section "Rigidity analyses") were placed between the binding nucleotides. (TIFF) [file pone.0179271.s015.tiff]
