# Supplementary material for: Ligand-mediated and tertiary interactions cooperatively stabilize the P1 region in the guanine-sensing riboswitch
Source: PLoS One. 2017 Jun 22;12(6):e0179271. doi: 10.1371/journal.pone.0179271 (PMC5480868; doi:10.1371/journal.pone.0179271)
Supplement: S1 Table — (PDF) [file pone.0179271.s016.pdf]

S1 Table: Occupation of experimentally determined Mg^{2+} binding sites[a]

Simulation system		Mg^{2+} binding sites							
		1[b]	2[b]	3[b]	4[b]	5[b]	6[b]	7[c]	8[c]
Gsw^{apt} 12 Mg^{2+} random initial position[d]		9	29	74	25	40	17	56	57
Gsw^{apt} 12 Mg^{2+}	1	10	27	99	28	88	47	75	55
	2	27	40	99	99	77	31	97	0
	3	94	74	89	73	95	51	5	8
Gsw^{apt} 20 Mg^{2+}	1	60	97	100	97	96	79	95	9
	2	69	100	97	88	94	30	78	14
	3	27	65	82	98	75	56	99	15
Gsw^{loop} 12 Mg^{2+}	1	32	13	99	93	5	74	100	5
	2	7	100	100	33	89	6	25	3
	3	25	10	80	14	16	4	85	5
Gsw^{loop} 20 Mg^{2+}	1	31	67	100	62	35	61	98	16
	2	54	73	100	80	74	90	63	57
	3	100	55	99	88	82	25	98	19

[a] In %. A Mg^{2+} ion occupied a binding site if it was closer than 5 Å to the binding site.

[b] $[Co(NH_3)_6]^{3+}$ position in crystal structures with PDB IDs 4FE5 (1) and 3RKF (2).

[c] Additional $[Co(NH_3)_6]^{3+}$ position found in crystal structure with PDB ID 3RKF (2).

[d] Mg^{2+} ions were randomly placed at least 10 Å away from the RNA.

References

1. Stoddard, C.D., Widmann, J., Trausch, J.J., Marcano-Velazquez, J.G., Knight, R. and Batey, R.T. (2013) Nucleotides adjacent to the ligand-binding pocket are linked to activity tuning in the purine riboswitch. *J. Mol. Biol.*, **425**, 1596-1611.
2. Buck, J., Wacker, A., Warkentin, E., Wöhnert, J., Wirmer-Bartoschek, J. and Schwalbe, H. (2011) Influence of ground-state structure and Mg^{2+} binding on folding kinetics of the guanine-sensing riboswitch aptamer domain. *Nucleic Acids Res.*, **39**, 9768-9778.
