# Supplementary material for: Ligand-mediated and tertiary interactions cooperatively stabilize the P1 region in the guanine-sensing riboswitch
Source: PLoS One. 2017 Jun 22;12(6):e0179271. doi: 10.1371/journal.pone.0179271 (PMC5480868; doi:10.1371/journal.pone.0179271)
Supplement: S2 Table — (PDF) [file pone.0179271.s017.pdf]

S2 Table: Core nucleotides taken into account for analysis[a]

Assignment	Nucleotide numbers
80% least fluctuating nucleotides	19-35, 37-47, 50-61, 65-78

[a] Determined from the MD simulations of Gsw^{apt} in the absence of Mg^{2+} ions as the 80% least fluctuating nucleotides. The core nucleotides were used for fitting the structures prior to the calculation of the RMSD (Table 1) and the RMSF (Fig. 2, S8 Fig), and for calculating the average RMSF in S4 Table.
