# Supplementary material for: Ligand-mediated and tertiary interactions cooperatively stabilize the P1 region in the guanine-sensing riboswitch
Source: PLoS One. 2017 Jun 22;12(6):e0179271. doi: 10.1371/journal.pone.0179271 (PMC5480868; doi:10.1371/journal.pone.0179271)
Supplement: S3 Table — (PDF) [file pone.0179271.s018.pdf]

S3 Table: Root mean square deviations of Gsw^{apt} and Gsw^{loop} [a]

Simulated system		Aptamer [b]	P1[c]	P2[c]	P3[c]	L2[c]	L3[c]
Gsw^{apt}	0 Mg^{2+}	3.0 ± 0.1	2.2 ± 0.2	1.4 ± 0.1	1.6 ± 0.1	1.5 ± 0.1	2.3 ± 0.2
	12 Mg^{2+}	2.9 ± 0.1	1.7 ± 0.1	1.1 ± 0.1	1.4 ± 0.1	1.1 ± 0.1	2.0 ± 0.1
	20 Mg^{2+}	2.3 ± 0.1	1.6 ± 0.1	1.2 ± 0.1	1.1 ± 0.1	1.3 ± 0.1	1.8 ± 0.1
Gsw^{loop}	0 Mg^{2+}	4.2 ± 0.2	2.5 ± 0.2	1.5 ± 0.1	2.0 ± 0.1	2.5 ± 0.1	2.8 ± 0.1
	12 Mg^{2+}	2.4 ± 0.1	2.1 ± 0.2	1.3 ± 0.1	1.2 ± 0.1	2.3 ± 0.1	2.4 ± 0.1
	20 Mg^{2+}	2.5 ± 0.1	1.8 ± 0.1	1.3 ± 0.1	1.2 ± 0.1	1.5 ± 0.1	2.5 ± 0.2

Simulated system		J1/2[c]	J2/3[c]	J3/1[c]
Gsw^{apt}	0 Mg^{2+}	1.3 ± 0.1	2.7 ± 0.1	1.2 ± 0.1
	12 Mg^{2+}	1.2 ± 0.1	3.2 ± 0.2	1.5 ± 0.2
	20 Mg^{2+}	1.2 ± 0.1	2.7 ± 0.2	0.8 ± 0.1
Gsw^{loop}	0 Mg^{2+}	1.6 ± 0.1	3.4 ± 0.2	2.0 ± 0.2
	12 Mg^{2+}	1.3 ± 0.0	2.8 ± 0.2	1.3 ± 0.1
	20 Mg^{2+}	1.2 ± 0.1	2.5 ± 0.1	1.0 ± 0.1

[a] In Å; given is the mean ± SEM calculated over the three trajectories for each simulated system; the first 50 ns of each trajectory were omitted for the calculations.
[b] The complete RNA (nucleotides 15-81). The RMSD was calculated after root mean-square fitting of the conformations on the initial conformation, considering only those 80% of the nucleotides ("core nucleotides", S2 Table) that show the lowest RMSF.
[c] RMSD of the respective substructure after root mean-square fitting of the conformations on the initial conformation, considering only the nucleotides of the substructure.
