# Supplementary material for: Ligand-mediated and tertiary interactions cooperatively stabilize the P1 region in the guanine-sensing riboswitch
Source: PLoS One. 2017 Jun 22;12(6):e0179271. doi: 10.1371/journal.pone.0179271 (PMC5480868; doi:10.1371/journal.pone.0179271)
Supplement: S4 Table — (PDF) [file pone.0179271.s019.pdf]

S4 Table: Root mean square fluctuations for Gsw^{apt} and Gsw^{loop} [a]

Gsw variant	0 Mg^{2+} [b]	12 Mg^{2+} [b]	20 Mg^{2+} [b]
Gsw^{apt}	1.70 ± 0.02	1.39 ± 0.01	1.18 ± 0.02
Gsw^{loop}	2.27 ± 0.03	1.34 ± 0.02	1.39 ± 0.02

[a] In Å. First, the mean ± SEM RMSF for each of the 80% least fluctuating nucleotides ("core nucleotides", Table S2) was calculated over three trajectories of 550 ns, after fitting onto the first structure taking into account only the core nucleotides; the first 50 ns of each trajectory were omitted. The values reported here are mean values ± SEM over these core nucleotides.

[b] Number of Mg^{2+} ions per RNA molecule.
