# Supplementary material for: Ligand-mediated and tertiary interactions cooperatively stabilize the P1 region in the guanine-sensing riboswitch
Source: PLoS One. 2017 Jun 22;12(6):e0179271. doi: 10.1371/journal.pone.0179271 (PMC5480868; doi:10.1371/journal.pone.0179271)
Supplement: S5 Table — (PDF) [file pone.0179271.s020.pdf]

S5 Table: Assignment of nucleotides to substructural parts of the Gsw aptamer domain[a]

Substructural part	Nucleotide numbers
P1	15-21, 75-81
P2	25-31, 39-45
P3	54-59, 67-72
L2	32-38
L3	60-66
J1/2	22-24
J2/3	46-53
J3/1	73,74

[a] According to Batey *et al.* (1).

1. Batey, R.T., Gilbert, S.D. and Montange, R.K. (2004) Structure of a natural guanine-responsive riboswitch complexed with the metabolite hypoxanthine. *Nature*, **432**, 411-415.
